# Supplementary material for: Referenced Single-Molecule Measurements Differentiate between GPCR Oligomerization States
Source: Biophys J. 2015 Nov 4;109(9):1798–806. doi: 10.1016/j.bpj.2015.09.004 (PMC4643199; doi:10.1016/j.bpj.2015.09.004)
Supplement: Document S1. Supporting Discussion and six figures [file mmc1.pdf]

SUPPORTING MATERIAL

Referenced single-molecule measurements differentiate between GPCR oligomerization states

Sarah L. Latty,* § James H. Felce,† § Laura Weimann, * Steven F. Lee,* Simon J. Davis,† ¶ and David Klenerman *¶

*Department of Chemistry, University of Cambridge, Lensfield Road, Cambridge CB2 1EW, United Kingdom; †Radcliffe Department of Clinical Medicine and Medical Research Council Human Immunology Unit, Weatherall Institute of Molecular Medicine, University of Oxford, Oxford OX3 9DS, United Kingdom. § These authors contributed equally. ¶ Corresponding authors.

SUPPORTING RESULTS

- **FIGURE S1 Diffusional behavior affects apparent stoichiometry**
- **FIGURE S2 Histograms of room temperature diffusion coefficients of fixed and unfixed puncta**
- **FIGURE S3 Expression levels of HaloTag constructs in HEK-293T and CHO-K1 cells**
- **FIGURE S4 Estimation of SNAP-tag/HaloTag labeling efficiency using antibody-staining**
- **FIGURE S5 Coincidence values obtained with a varying distance criterion**
- **FIGURE S6 The dependence of coincidence on labeling efficiency for monomers and dimers**

SUPPORTING DISCUSSION

SUPPORTING REFERENCES

SUPPORTING RESULTS

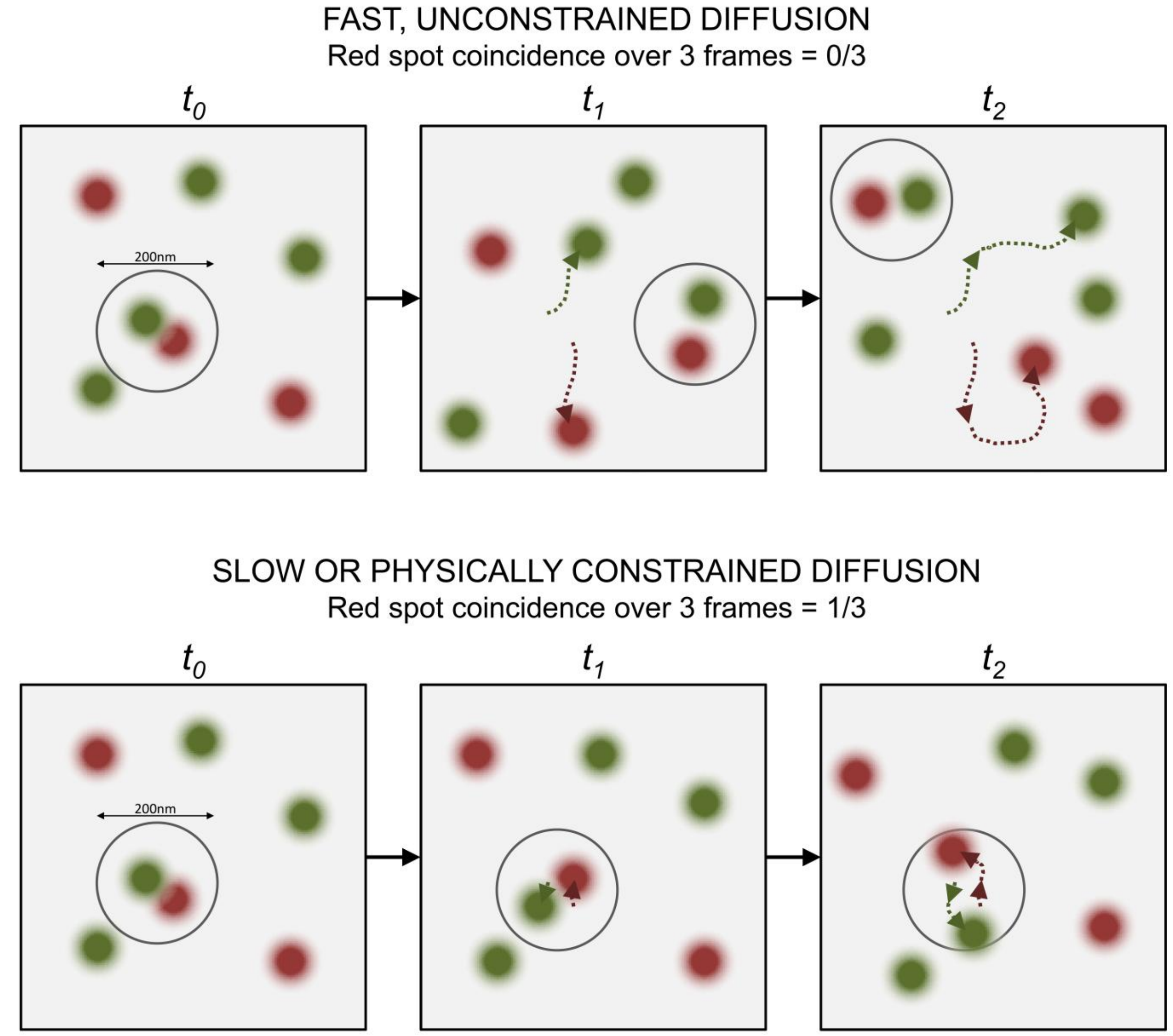

FIGURE S1 Diffusional behavior affects apparent stoichiometry: proteins that have equivalent global distributions within a membrane can have different levels of coincidence if they have different diffusional behavior. Red and green spots represent proteins labeled in two colors. Circles indicate the resolution of diffraction-limited microscopy (typically 200-300nm) within which two spots will be colocalized. Diffusional paths for the starting colocalized pair are shown as broken arrows. For fast diffusing proteins (top row), the number of spots within the colocalization distance of a spot of the opposite color will remain constant at any given time-point (t_0, t_1, t_2, *etc*.). However, for a spot to be identified as coincident, it must remain colocalized with the same partner for a defined number of frames. Although there will be colocalized spots at any given time-point, very few will remain colocalized for sufficient time to be identified as coincident. Hence, coincidence will be low for rapidly diffusing proteins. Proteins that diffuse more slowly or are physically constrained in their diffusion (bottom row) are more likely to remain within the colocalization distance of the same partner for multiple frames, and so coincidence will be higher than for more rapidly diffusing proteins at the same density. Fixation removes the variable of diffusion, and all proteins with the same stoichiometry and density will exhibit the same level of coincidence. This allows comparison to controls of known stoichiometry without the risk of diffusion inadvertently affecting the comparisons.

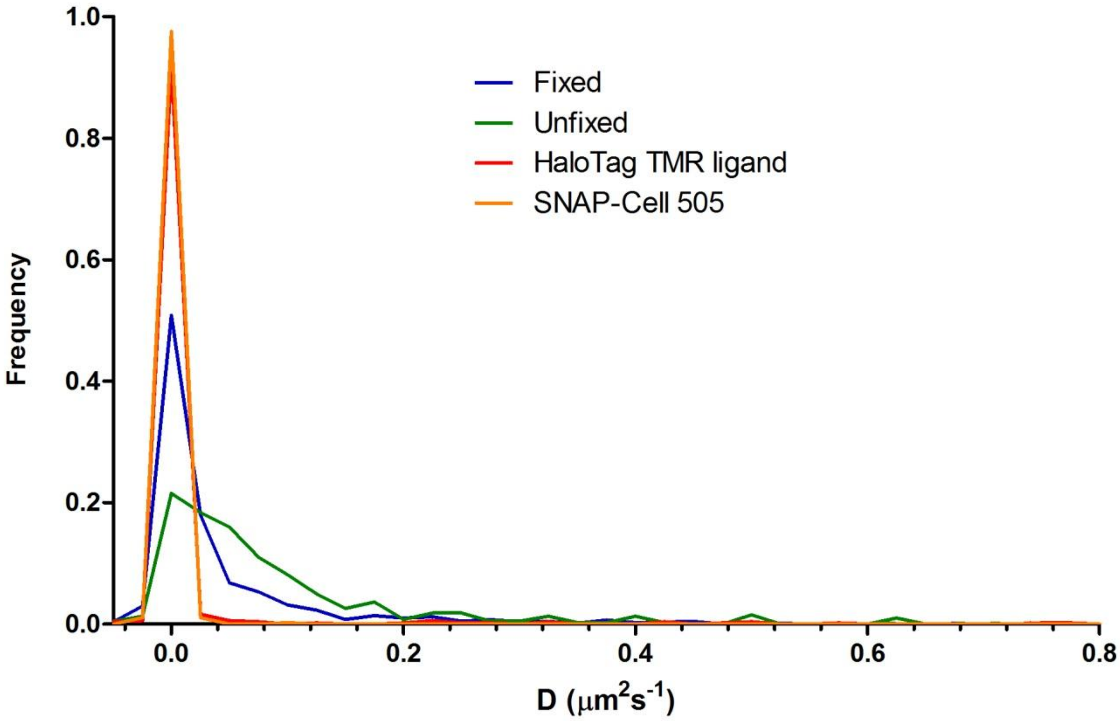

FIGURE S2 Histograms of diffusion coefficients of tagged CD86 on unfixed and fixed cells. Diffusional data for TMR and SNAP-Cell 505 fluorophores adsorbed to glass is also displayed. Diffusion coefficients were calculated using a Mean-Square Displacement (MSD) analysis. Assuming ‘random walk’ receptor motion a linear function was utilized to relate MSD to Brownian diffusion (1). Apparently negative diffusion coefficients should be interpreted as having a diffusion coefficient of zero within experimental error. Such coefficients arise by chance for some molecules undergoing very small motions as MSD plots can produce a negative slope i.e. the molecule moves further at short times than long times. Across all collected puncta for all fixed proteins, 5.9% had a diffusion coefficient greater than $0.2 \mu m^2 s^{-1}$, which is the limit above which spots will have moved from their original 300nm-diameter area ($0.07 \mu m^2$) within the 10 frame (350ms) period. In unfixed cells, 11.5% of CD86 trajectories had a diffusion coefficient greater than $0.2 \mu m^2 s^{-1}$. The difference in diffusion between fixed and unfixed samples was tested using a Kolmogorov-Smirnov comparison and found to be significant to $p<0.001$ with a maximum difference of cumulative distributions (D) of 0.37. Fixed trajectories had a reduced difference in diffusion relative to adsorbed fluorophores (D=0.55) compared to unfixed puncta (D=0.80). The fixation strategy, therefore, is effective in immobilizing the majority of tagged proteins.

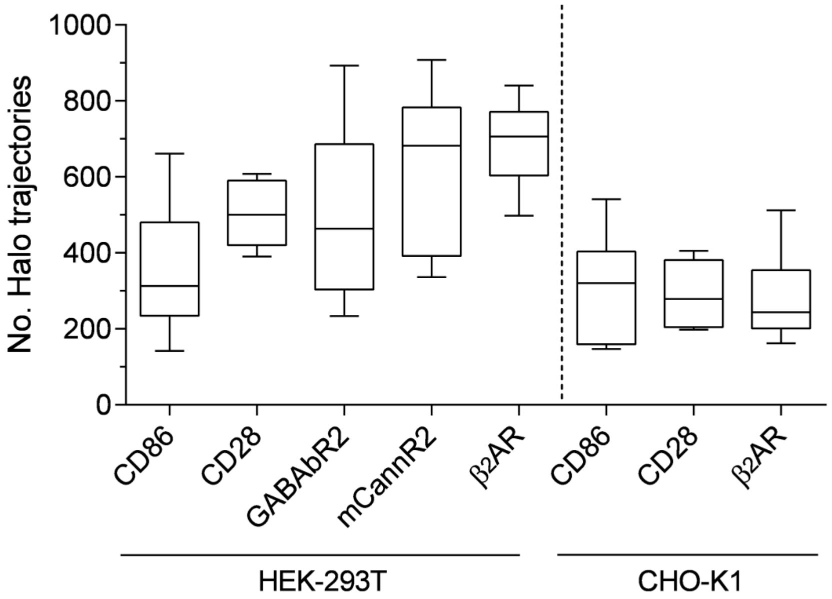

FIGURE S3 Expression levels of HaloTag constructs in HEK-293T and CHO-K1 cells. Data are shown as minimum to maximum box-and-whisker plots.

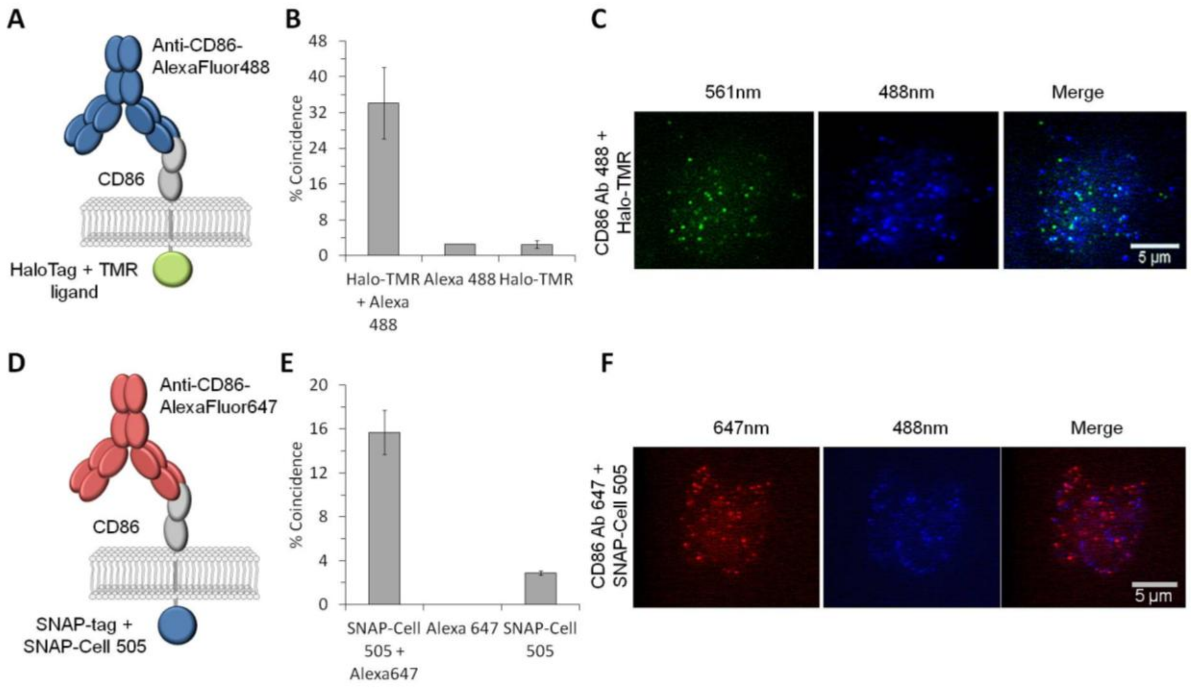

FIGURE S4 Estimation of SNAP-tag/HaloTag labeling efficiency in HEK-293T cells using antibody-staining. (**A** & **D**) Representations of the strategy used in each case: anti-CD86 antibody conjugated to either AlexaFluor 647 or AlexaFluor 488 was used to provide a second label for CD86 fused to either HaloTag or SNAP-tag. Antibodies were used at 100 μg/ml to ensure saturating, monovalent binding. (**B** & **E**) Coincidence values for spots in the two relevant colors. Coincidence is given relative to antibody spots, i.e. coincidence represents the percentage of antibody spots for which an associated tag ligand spot was detected. Images were collected on cells labeled with both antibody and tag ligand, antibody only, and tag ligand only. Bars are mean ± SEM. (**C** & **F**) Representative examples of cells labeled with both antibody and tag ligand.

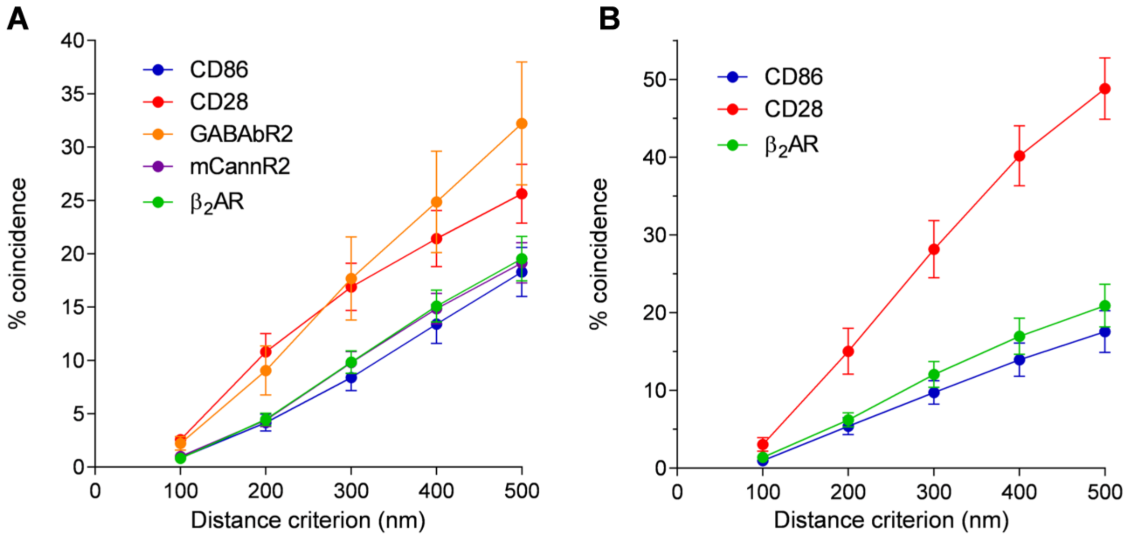

FIGURE S5 Coincidence values obtained with a varying distance criterion. The overall assignment of stoichiometry for GPCRs relative to CD86 and CD28 controls is unaffected by the distance criterion used to define coincidence in both HEK-293T (**A**) and CHO-K1 (**B**) cells. Bars are mean ± SEM.

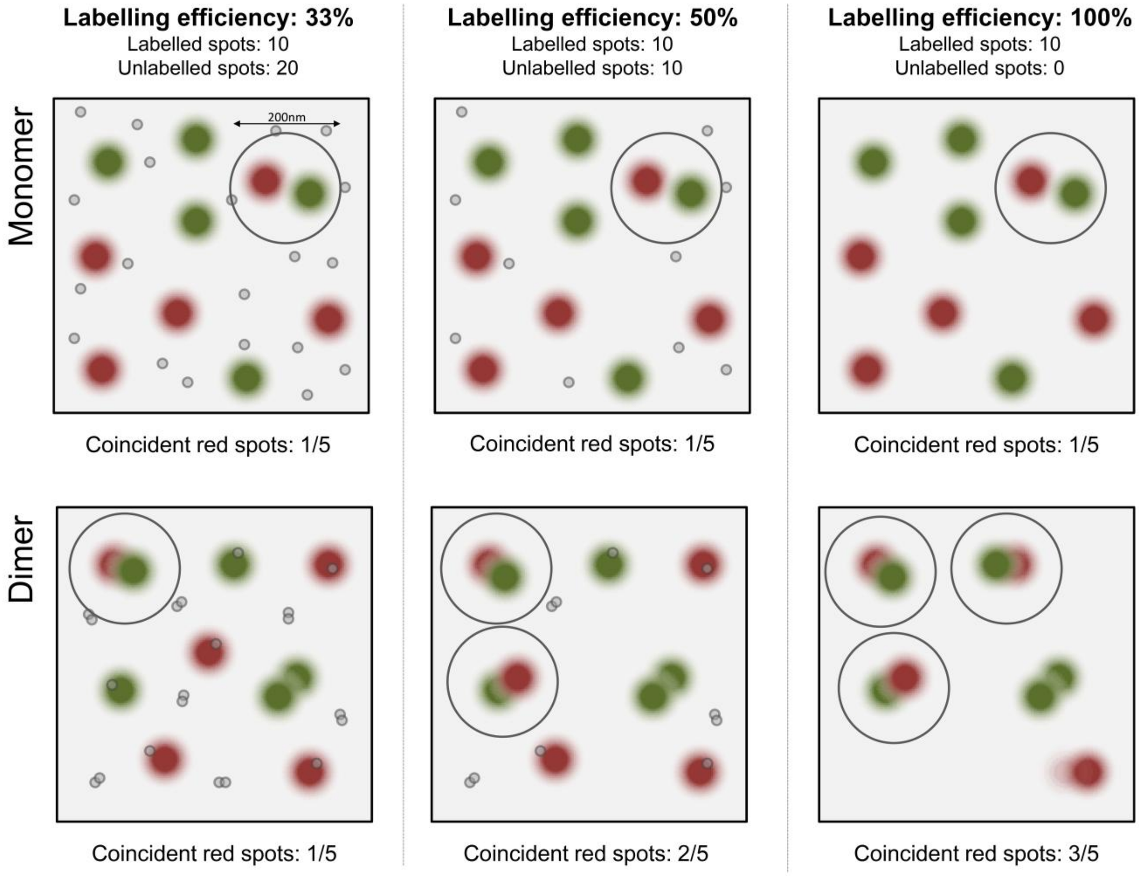

FIGURE S6 The dependence of coincidence on labeling efficiency for monomers and dimers. Proteins are shown as a schematic of labeled (red and green) and unlabelled (grey) spots at varying labeling efficiencies. Circles indicate the resolution of diffraction-limited microscopy (typically 200-300nm) within which two spots will be colocalized. If the total density of labeled proteins is constant, the coincidence of monomeric proteins is independent of labeling efficiency (top row). This is because coincidence of monomers is the result only of random colocalization of labeled proteins within the diffraction limit of the detection method. If labeling efficiency increases but the density of labeled spots remains constant, there will be fewer unlabeled proteins but the coincidence of the labeled proteins will not change. In contrast, the coincidence of dimers is dependent on labeling efficiency as unlabeled proteins compete for position within dimers and hence reduce coincidence. Thus, as labeling efficiency increases, coincidence of dimers also increases (bottom row).

SUPPORTING DISCUSSION

Derivation of the relationship between labeling efficiency, relative expression level, and coincidence.

Let:
n = Fraction all CD28 protomers SNAP-tagged (labeled and unlabeled)
m = Fraction all CD28 protomers Halo-tagged (labeled and unlabeled)
p = SNAP-tag labeling efficiency
q = HaloTag labeling efficiency
f = Ratio of SNAP(labeled):Halo(labeled)
c = Fraction of CD28 dimers containing both HaloTag(labeled) and SNAP-tag(labeled) (*i.e.* coincident fraction)

∴
$$Fraction\ CD28\ protomers\ with\ Halo(labeled) = mq$$
$$Fraction\ CD28\ protomers\ with\ Halo(unlabeled) = m(1-q)$$
$$Fraction\ CD28\ protomers\ with\ SNAP(labeled) = np$$
$$Fraction\ CD28\ protomers\ with\ SNAP(unlabeled) = n(1-p)$$

∴
$$Fraction\ dimers\ with\ 2 \times Halo(labeled) = m^2q^2$$
$$Fraction\ dimers\ with\ 1 \times Halo(labeled) +\ 1 \times SNAP(labeled) = 2mqnp$$
$$Fraction\ dimers\ with\ 1 \times Halo(labeled) +\ 1 \times SNAP(unlabeled) = 2mqn(1-p)$$
$$Fraction\ dimers\ with\ 1 \times Halo(labeled) +\ 1 \times Halo(unlabeled) = 2mqm(1-q)$$

$$c = \frac{Fraction\ dimers\ with\ 1 \times Halo(labelled) +\ 1 \times SNAP(labelled)}{Fraction\ dimers\ with\ at\ least\ 1\ \times Halo(labelled)}$$

$$= \frac{2mqnp}{(m^2q^2) + (2mqnp) + \left(2mqn(1-p)\right) + \left(2mqm(1-q)\right)}$$

$$= \frac{2np}{2n + 2m - mq}$$

$$Since\ n = 1 - m$$

$$c = \frac{2p(1-m)}{2 - mq}$$

$$\therefore if\ p = q$$

$$p = \frac{2c}{2 + cm - 2m}$$

$$\therefore if\ 2p = q$$

$$p = \frac{c}{1 + cm - m}$$

$$Ratio\ SNAP(labeled){:}\ Halo(labeled) = \frac{Fraction\ CD28\ protomers\ with\ SNAP(labeled)}{Fraction\ CD28\ protomers\ with\ Halo(labeled)}$$

$$\therefore f = \frac{np}{mq} = \frac{np}{(1-n)q} = \frac{np}{q-nq}$$

$$\therefore n = \frac{qf}{qf+p}$$

$$\therefore m = 1 - \left(\frac{qf}{qf+p}\right)$$

$$\therefore if\ p = q$$

$$m = 1 - \left(\frac{f}{(1+f)}\right)$$

$$\therefore if\ 2p = q$$

$$m = 1 - \left(\frac{2f}{(1+2f)}\right)$$

For CD28 in HEK-293T cells
c = observed coincidence = 0.17
f = observed SNAP:Halo ratio = 3.23
$\therefore if\ 2p = q$ $\boldsymbol{m = 0.134}$ $\boldsymbol{p = 19.1\%}$ $\boldsymbol{q = 38.3\%}$
$\therefore if\ p = q$ $\boldsymbol{m = 0.236}$ $\boldsymbol{p = 21.7\%}$ $\boldsymbol{q = 21.7\%}$

For CD28 in CHO-K1 cells
c = observed coincidence = 0.28
f = observed SNAP:Halo ratio = 3.27
$\therefore if\ 2p = q$ $\boldsymbol{m = 0.133}$ $\boldsymbol{p = 30.9\%}$ $\boldsymbol{q = 61.9\%}$
$\therefore if\ p = q$ $\boldsymbol{m = 0.234}$ $\boldsymbol{p = 36.3\%}$ $\boldsymbol{q = 36.3\%}$

For CD28 in HEK-293T cells using measured labeling efficiencies
p = measured SNAP labeling efficiency = 0.16
q = measured Halo labeling efficiency = 0.33
f = observed SNAP:Halo ratio = 3.23
$\therefore m = 0.1305$
$c = \frac{2p(1-m)}{2-mq} = \frac{2\times0.16\times(1-0.1305)}{2-(0.1305\times0.33)} = \boldsymbol{0.142}$ **= 14.2%**

This is close to the observed coincidence of 16.9%.

SUPPORTING REFERENCES

1. Weimann, L., K. A. Ganzinger, J. McColl, K. L. Irvine, S. J. Davis, N. J. Gay, C. E. Bryant, and D. Klenerman. 2013. A Quantitative Comparison of Single-Dye Tracking Analysis Tools Using Monte Carlo Simulations. PLoS One 8.
