# Supplementary material for: Referenced Single-Molecule Measurements Differentiate between GPCR Oligomerization States
Source: Biophys J. 2015 Nov 4;109(9):1798–806. doi: 10.1016/j.bpj.2015.09.004 (PMC4643199; doi:10.1016/j.bpj.2015.09.004)
Supplement: Document S2. Article plus Supporting Material [file mmc2.pdf]

Article

Referenced Single-Molecule Measurements Differentiate between GPCR Oligomerization States

Sarah L. Latty,[1] James H. Felce,[2] Laura Weimann,[1] Steven F. Lee,[1] Simon J. Davis,[2,*] and David Klenerman[1,*]
[1]Department of Chemistry, University of Cambridge, Cambridge, United Kingdom; and [2]Radcliffe Department of Clinical Medicine and Medical Research Council Human Immunology Unit, Weatherall Institute of Molecular Medicine, University of Oxford, Oxford, United Kingdom

ABSTRACT The extent to which *Rhodopsin* family G-protein-coupled receptors (GPCRs) form invariant oligomers is contentious. Recent single-molecule fluorescence imaging studies mostly argue against the existence of constitutive receptor dimers and instead suggest that GPCRs only dimerize transiently, if at all. However, whether or not even transient dimers exist is not always clear due to difficulties in unambiguously distinguishing genuine interactions from chance colocalizations, particularly with respect to short-lived events. Previous single-molecule studies have depended critically on calculations of chance colocalization rates and/or comparison with unfixed control proteins whose diffusional behavior may or may not differ from that of the test receptor. Here, we describe a single-molecule imaging assay that 1) utilizes comparisons with well-characterized control proteins, i.e., the monomer CD86 and the homodimer CD28, and 2) relies on cell fixation to limit artifacts arising from differences in the distribution and diffusion of test proteins versus these controls. The improved assay reliably reports the stoichiometry of the *Glutamate*-family GPCR dimer, γ-amino butyric acid receptor b2, whereas two *Rhodopsin*-family GPCRs, β_2-adrenergic receptor and mCannR2, exhibit colocalization levels comparable to those of CD86 monomers, strengthening the case against invariant GPCR oligomerization.

INTRODUCTION

Advances in fluorescence microscopy now allow the routine detection and tracking of individual fluorescently labeled proteins at the cell surface, e.g., using techniques such as single-molecule total internal reflection fluorescence (smTIRF) microscopy. Single-molecule imaging is particularly attractive for the study of protein stoichiometry, as it allows direct visualization of protein-protein interactions, obviating the need for interpreting stoichiometry from indirect assays.

Two general approaches have been taken. The first is based on labeling with a single fluorophore and using photobleaching-step counting or intensity analysis to determine protein stoichiometry (1–4), which can be combined with selective photobleaching to achieve improved resolution (5). The second approach uses the labeling of proteins with two spectrally distinct fluorophores and detecting colocalization events to count the number of associated molecules (6,7). In both cases, there are issues that need to be addressed concerning the efficiency of protein labeling and how this is taken into account in the analysis, and there is also the problem of apparent association, which arises due to two molecules being in close spatial proximity by chance. The likelihood of chance association is usually estimated from the protein density assuming that diffusion is random, which may or may not be the case (8,9).

An alternative approach is to compare the behavior of the protein of interest with that of proteins of known stoichiometry, i.e., monomeric and dimeric controls. This establishes the maximum and minimum levels (the dynamic range) of signal expected in the experiment, taking account of labeling efficiency and nonrandom diffusion. This approach proved to be especially useful for the single-molecule video-based analysis of the stoichiometry of the T-cell receptor (5), wherein the cytoskeleton contributed a large fraction of the measured coincidence by apparently constraining receptor diffusion. A potential difficulty exists, however, in that the control proteins may have intrinsically different diffusive behavior versus the proteins of interest, in which case direct comparisons utilizing video-based molecule tracking could be problematic. The type of problem that could arise is illustrated in Fig. S1 in the Supporting Material. Such effects can be avoided, however, if the proteins are fixed, as the variable of diffusion is removed and an effective snapshot of receptor oligomerization propensity can be obtained (Fig. S1).

A field of inquiry in which single-molecule microscopy has been especially influential is the analysis of G-protein-coupled receptor (GPCR) stoichiometry, which is arguably the single most contentious aspect of their biology. Smaller-family GPCRs, e.g., the *Glutamate* family (10), are known to dimerize through large N- and C-terminal

Submitted February 20, 2015, and accepted for publication September 8, 2015.

*Correspondence: dk10012@cam.ac.uk or simon.davis@imm.ox.ac.uk

This is an open access article under the CC BY license (http://creativecommons.org/licenses/by/4.0/).

Sarah L. Latty and James H. Felce contributed equally to this work.

Editor: Antoine van Oijen.

© 2015 The Authors

0006-3495/15/11/1798/9

http://dx.doi.org/10.1016/j.bpj.2015.09.004

CrossMark

domains, but the resting stoichiometries of the most numerous and pharmacologically important GPCRs, i.e., the receptors comprising the *Rhodopsin* family, are vigorously disputed. This is an important matter, because in the few studies in which it was examined in single-molecule fluorescence experiments, receptor agonists had no discernible effect on receptor stoichiometry (reviewed by Kasai and Kusumi (11)). This suggests that any requirement for receptor oligomerization as such might already be met before activation of the receptor. Although it is known that several *Rhodopsin* family receptors can function autonomously, i.e., as isolated monomers (12–14), they are nonetheless proposed to form functionally distinct dimeric and oligomeric complexes. Early claims for constitutive *Rhodopsin*-family GPCR oligomerization were based on resonance energy transfer (RET) measurements and coimmunoprecipitation analyses, as well as on ostensibly cooperative receptor behavior, but these conclusions have often been disputed (15–18). More recently, single-molecule measurements have challenged the general model of constitutive dimerization, after consistently failing to demonstrate the required levels of receptor oligomerization in transfected and native cells (3,6,19), with just one exception (2). These studies have instead reported levels of transient receptor association above those expected by chance, leading to the constitutive dimer model being supplanted by another comprised of transient dimerization (20). It is uncertain, however, whether the measured signals were the product of genuine interactions or instead reflected difficulties in calculating the nonspecific background signal. The real extent of GPCR dimerization is therefore unresolved.

In this study, we circumvent the issues inherent in single-molecule imaging-based stoichiometric analysis by 1) utilizing well-characterized control proteins, i.e., the monomer CD86 (16) and the homodimer CD28 (21), to establish the dynamic range of the experiment, and 2) relying on cell fixation to limit artifacts arising from differences in the distribution and diffusion of test proteins versus our control proteins. We show that the improved assay reliably reports the stoichiometry of the *Glutamate*-family GPCR dimer γ-amino butyric acid receptor b2 (GABAbR2), whereas two *Rhodopsin*-family GPCRs, β_2-adrenergic receptor (β_2AR) and mCannR2, exhibit colocalization levels comparable to those of CD86, arguing against invariant GPCR oligomerization.

MATERIALS AND METHODS

Construct design and molecular cloning

Two expression vectors, pHRI-SNAP and pHRI-Halo, were generated from the previously described pHRI-Citrine$_3$ vector (7) by replacement of the three constituent *Citrine* genes with those encoding either SNAP-tag or HaloTag using flanking *Bam*HI and *Not*I restriction endonuclease sites. The new vectors encoded the SNAP-tag or HaloTag proteins at the C-terminus of proteins of interest encoded by genes inserted into the upstream multiple cloning site. In both pHRI vectors, expression of inserted genes is under the control of the ecdysone-dependent minimal heat-shock promoter from the pCFB-EGSH vector (Agilent Technologies, Santa Clara, CA) such that gene expression is limited to ~2000–4000 per cell in the absence of ecdysone or equivalent analogs. Genes encoding CD86, CD28, GABAbR2, mCannR2, and β_2AR were subcloned from preexisting constructs (described in James et al. (16)) by ligation into pHRI-SNAP or pHRI-Halo after digestion with the following restriction endonucleases: CD86, *Mlu*I + *Age*I; CD28, *Eco*RI + *Bam*HI; GABAbR2, *Mlu*I + *Kpn*I; mCannR2, *Mlu*I + *Bam*HI; and β_2AR, *Mlu*I + *Bam*HI. The integrity of all constructs was confirmed by direct sequencing.

Cell culture and plating

Human embryonic kidney (HEK)-293T cells were grown in DMEM supplemented with 10% fetal calf serum, 1% L-glutamine, and 1% antibiotics (Life Technologies, Carlsbad, CA). Chinese hamster ovary (CHO)-K1 cells were grown in Dulbecco's modified Eagle's medium (DMEM) supplemented with 10% fetal calf serum, 1% antibiotics, 1% L-glutamine, 1% sodium pyruvate, and 2% supplement solution (70 mg adenosine, 70 mg cytidine, 70 mg guanosine, 70 mg uridine, 24 mg thymidine, 20 mg asparagine, 20 mg glutamic acid, and deionized H_2O to a 200 mL final volume). HEK-293T/CHO-K1 cells were plated in their respective media 24 h before transfection in six-well plates at 3.0×10^5 cells/mL, 2 mL/well.

Transfection

Constructs were cotransfected at a given ratio of the vectors encoding the HaloTag- and SNAP-tag-labeled proteins using GeneJuice (Novagen, Madison, WI) as per the manufacturer's instructions. The ratio of tagged constructs and the incubation times were optimized for each gene to achieve densities of 100–1000 HaloTag spots per cell, and a SNAP-tag/HaloTag ratio of 1–6, as given in Table 1.

Labeling

A stock solution of HaloTag TMR Ligand (Promega, Madison, WI) was diluted in supplemented DMEM to a final concentration of 5 nM. After incubation posttransfection, all medium was removed from the cells and replaced with supplemented DMEM containing 5 nM HaloTag TMR Ligand (Promega) and 5 μM SNAP-Cell 505-Star (New England Biolabs, Ipswich, MA) before incubation for 30 min at 37°C. Cells were then washed with 3 $\times$ 1 mL medium and incubated for an additional 30 min in 1 mL of medium before a final wash with 3 $\times$ 1 mL medium.

Sample preparation for microscopy

Microscope coverslips were plasma cleaned (PDC-002, Harrick Plasma, Ithaca, NY) in an argon atmosphere for 30 min before subsequent coating

TABLE 1 Transfection conditions used to obtain optimal conditions for imaging

Protein	Cell Line	Halo-Tagged Construct (μg)	SNAP-tagged Construct (μg)	Posttransfection Incubation (h)
CD86	HEK-293T	0.975	0.025	7.0
CD28	HEK-293T	0.975	0.025	5.0
mCannR2	HEK-293T	0.975	0.150	4.0
GABAbR2	HEK-293T	0.975	0.080	42.0
β_2AR	HEK-293T	1.500	0.300	4.0
CD86	CHO-K1	0.975	0.175	20.0
CD28	CHO-K1	0.975	0.175	48.0
β_2AR	CHO-K1	0.975	0.080	18.5

with poly-L-lysine-grafted polyethyleneglycol (PLL-g-PEG, SuSoS) for 45 min. Slides were then washed in duplicate with filtered (0.22 μm Millex-GP syringe filter unit, Millipore, Billerica, MA) phosphate-buffered saline (PBS, Life Technologies). After labeling and washing, cells were mechanically removed and centrifuged at 2000 rpm for 2 min. The resultant supernatant was removed and cells were resuspended in 200 μL DMEM before being added onto coated cover slides. The plated cells were then allowed to settle at 37°C for 20 min. Medium on cover slides was then replaced with 4% formaldehyde solution (16% w/v stock solution (Thermo Fisher Scientific, Waltham, MA) diluted to 4% with PBS). The formaldehyde solution was left on the plated cells for 1 h at room temperature before replacement with PBS immediately preceding imaging.

smTIRF microscopy experimental set-up

The samples were imaged using smTIRF microscopy. Solid-state lasers operating at 488 nm (Cyan, Petaluma, CA, and Spectra Physics, Santa Clara, CA) and 561 nm (Excelsior-561-20-CDRH, Spectra Physics) were used for the imaging, using a quarter-wave plate to ensure that the beams were circularly polarized. Both beams entered the microscope on the edge of the back focal plane of a 1.45 NA TIRF objective (60× Plan Apo TIRF, NA 1.45, Nikon, Tokyo, Japan) mounted on a Nikon TE2000-U microscope. A dichroic mirror (490575DBDR, Omega Optical, Brattleboro, VT) separated the collected fluorescence from the returning TIR beam. The fluorescence signal was then split into red and yellow components (585 DXLR, Omega Optical) and filtered using Dual-ViewTM (Optical Insights, Suwanee, GA) mounted filters.

Images were acquired on an electron-multiplying charge-coupled device equipped with a dual-view imaging system (Cascade II + DV2:512, Princeton Instruments, Trenton, NJ), with the device split such that each color was recorded on one-half of the device (−70°C; dichroic: DV2 FF562-Di03, Semrock, Rochester, NY). Data acquisition was performed using Micromanager Software, version 1.4.13. Image stacks were recorded over 400 frames with exposure set at 35 ms. The operating power densities for the 488 nm and 561 nm lasers used to acquire data sets were 1.66 W cm^{-2} and 5.19 W cm^{-2}, respectively.

Colocalization analysis

Spots were identified from image stacks using a previously published custom algorithm (22). Briefly, fluorescent puncta are superlocalized in each frame of an input TIFF file after calculation of their respective intensity-weighted centers of mass (centroids). Spatial coordinates are then connected in subsequent frames with their nearest neighbors so that analysis can be performed on molecules that are fluorescent for multiple frames. Fluorescence intensity thresholds are determined such that an optimum number of puncta are detected in both spectral channels before implementation of the colocalization analysis. This was empirically determined to be five standard deviations above the mean background for the HaloTag TMR channel and three standard deviations over the mean background for the SNAP-Cell 505-Star signal in the experiments described here.

Diffusional analysis

As reported by others (23), we found that a small fraction of molecules (typically <6%) are still mobile after fixation of the cells ($D \geq 0.2\ \mu m^2\ s^{-1}$). Diffusion coefficients for individual proteins were determined from the measurement of the position of the puncta with time to determine what fraction are mobile and immobile using a mean-square displacement (MSD) analysis (22). This small fraction of mobile events was prevented from contributing to the coincidence calculation by collecting multiple frames and applying a minimum frame criterion to the coincidence metric (see Colocalization criterion below).

Positional and registration accuracy

Our estimate of the positional accuracy is based on the standard deviation of the temporal positions of our detected fluorophores. Our mean positional accuracy was found to be 44 ± 22 nm (mean ± SD) for the yellow channel (HaloTag TMR ligand) and 38 ± 21 nm for the blue channel (SNAP-Cell 505-Star). The image registration error refers to the alignment of the two color channels (offset by 256 pixels horizontally and 0 pixels vertically). To minimize registration error, an alignment procedure was followed involving use of a custom-made regularly spaced grid with ion-beam-etched holes in gold on glass. White-light illumination of the grid allowed adjustment of dual-view optics such that overlap between red and green images was optimal. By adjusting the holes such that they appeared overlaid, a calculation of the image registration error was undertaken using a bespoke MATLAB-implemented algorithm. The mean image registration error for the obtained data sets was 158 nm.

Colocalization criterion

The colocalization criterion can be calculated by summing the root mean square of the calculated positional accuracies in each channel, given by the formula

$$\sigma^t = \sqrt{\sigma^{\text{red}2} + \sigma^{\text{green}2}}.$$

To obtain a 90% probability of colocalization, we apply a distance threshold of 1.65 σ^t (24). Adding our value for the image registration accuracy, this gives us a colocalization distance value of 217 nm. To allow for this, colocalization fractions are reported at 300 nm. This ensures that the values we report are most likely to be true colocalization events. Selection of a 300 nm threshold also ensures that any error in our accuracy estimates will not influence the reliability of the colocalization values. Our conclusions were unaffected by the distance criteria used, although the coincidence values increased as the distance criterion increased (Fig. S5), as expected.

A minimum-frame (MF) criterion was also applied to the data. The MF value is a function incorporated into the colocalization program, which is defined as the minimum number of frames required for two fluorophores to remain associated before being classed as coincident and removes the contribution of noise from the data. The MF criterion also prevents the small fraction of mobile spots from contributing to the analysis, since the probability of two nonassociated fluorophores remaining within the colocalization distance for multiple frames is low. In this case, the MF value was set to 10. Assigning an MF value of 10 did not impede the detection of coincidence, as determined using a subset of data from four cells expressing GABAbR2 (1653 HaloTag spots and 4381 SNAP-tag spots in total), which gave a consistent actual coincidence value of 15.6% using MF values between 1 and 10.

Calculation of coincidence

The coincidence value was calculated relative to the total number of fluorescent HaloTag spots for each cell. The total number of spots for which coincident SNAP-tag and HaloTag fluorescence was detected, $N_{\text{SNAP-Halo}}$, was divided by the sum of the coincident and noncoincident Halo Tag spots, $N_{\text{SNAP-Halo}}+ N_{\text{Halo}}$ to give the reported coincidence value using the formula

$$\text{Coincidence value} = N_{\text{SNAP-Halo}} / (N_{\text{SNAP-Halo}} + N_{\text{Halo}}).$$

The coincidence value for each cell was then used to calculate the mean coincidence per cell and mean ± SE values.

Estimation of labeling efficiency

To estimate labeling efficiency directly, CD86-HaloTag- or CD86-SNAP-tag-transfected HEK-293T cells were stained with mouse anti-CD86 monoclonal IgG1 (clone BU63, VWR International, Radnor, PA). The antibody was first conjugated to either AlexaFluor488 or AlexaFluor647 using antibody-labeling kits (Life Technologies), after which the conjugation efficiency was assessed spectroscopically. The mean labeling efficiency for AlexaFluor488 was 3.1 dye molecules/antibody, and 2.9 dye molecules/antibody for AlexaFluor 647. Cell staining was performed after labeling with SNAP-tag and HaloTag ligands at an antibody concentration of 100 μg/mL in ice-cold PBS for 45 min. Cells were then washed three times with 1.5 mL cold PBS before being transferred to cover slides and fixed as described above. Coincidence was then measured between the fluorescent antibody spots and SNAP-tag or HaloTag spots as an estimate of labeling efficiency (Fig. S4). HaloTag labeling efficiency was in this way estimated to be ~33%, and SNAP-tag labeling efficiency ~16%.

Calculation of labeling levels for CD28 based on the coincidence level

It is possible also to calculate the labeling efficiencies for SNAP-tag and HaloTag that would be required to achieve a certain level of CD28 coincidence at a given ratio of fluorescent SNAP-tag:HaloTag spots. This allows us to independently estimate the labeling efficiencies and to confirm that the efficiencies measured using antibody coincidence are consistent with observed CD28 coincidence.

Two factors determine the coincidence level relative to fluorescent HaloTag spots: the labeling efficiency of both SNAP-tag and HaloTag and the relative numbers of each tag at the cell surface. If we let the fraction of CD28 protomers fused to SNAP-tag be n and the fraction fused to HaloTag m, then $n + m = 1$. If the labeling efficiencies of SNAP-tag and HaloTag are defined as p and q, respectively, then the expected coincidence value relative to fluorescent HaloTag spots (c) is given by the equation (see the Supporting Discussion for the full derivation)

$$\text{HaloTag coincidence} = c = \frac{2np}{2n + 2m - mq}.$$

n and m can be determined using the labeling efficiencies p and q as well as the observed ratio of fluorescent SNAP-tag/HaloTag spots, given by f, through the relationship

$$f = \frac{np}{mq}.$$

Using the known values for c and f, it is therefore possible to calculate the expected values for p and q. To do this, it is necessary to estimate the relative efficiencies of SNAP-tag and HaloTag labeling. In the simplest model, the labeling efficiencies are assumed to be equal, and hence, $p = q$. In this case,

$$p = q = \frac{2c}{2 + cm - 2m}$$

and

$$m = 1 - \left(\frac{f}{(1+f)}\right)$$

(see the Supporting Discussion for the full derivation). For CD28 in HEK-293T cells, $c = 0.17$ and $f = 3.23$, which suggests a labeling efficiency of 22% for both SNAP-tag and HaloTag if both are assumed to be equal. Similarly, for CD28 in CHO-K1 cells, $c = 0.28$ and $f = 3.27$, indicating labeling efficiencies of 36%.

The labeling efficiency values can also be calculated assuming that HaloTag labeling efficiency is twice that of SNAP-tag labeling, as indicated by direct measurement of labeling efficiency through antibody staining. In this model, $2p = q$, and hence,

$$p = \frac{c}{1 + cm - m}$$

and

$$m = 1 - \left(\frac{2f}{(1+2f)}\right)$$

(see the Supporting Discussion for the full derivation). Using the same values for c and f as above, this model gives an estimate of 19% SNAP-tag and 38% Halo-Tag labeling in HEK-293T cells, and 31% SNAP-tag and 62% Halo-Tag labeling in CHO-K1 cells.

The labeling efficiency values measured using antibody labeling ($p = 0.16$, $q = 0.33$) in HEK-293T cells can be combined with the observed SNAP-tag/HaloTag ratio ($f = 3.23$) to determine expected coincidence. This gives an expected coincidence of 14.2%, which is close to the observed value of 16.9%, indicating that our estimates of labeling efficiency are reasonable.

Estimation of sensitivity

The minimum level of dimerization detectable in this assay can be estimated by determining the minimum difference between the sample mean coincidence and the mean coincidence for CD86 required for statistical significance taking into account the measured mean $\pm$ SE values for each sample, which is expressed as the t-statistic,

$$t = \frac{\overline{x}_{\text{sample}} - \overline{x}_{\text{CD86}}}{\sqrt{\text{mean} \pm \text{SE}^2_{\text{sample}} + \text{mean} \pm \text{SE}^2_{\text{CD86}}}}.$$

This can then be expressed as percent coincidence by normalization against the mean difference between CD86 and CD28, which represent 0 and 100% dimerization, respectively.

$$\text{Fraction dimers required for detection} = \frac{\overline{x}_{\text{sample}} - \overline{x}_{\text{CD86}}}{\overline{x}_{\text{CD28}} - \overline{x}_{\text{CD86}}} = \frac{t\sqrt{\text{mean} \pm \text{SE}^2_{\text{sample}} + \text{mean} \pm \text{SE}^2_{\text{CD86}}}}{\overline{x}_{\text{CD28}} - \overline{x}_{\text{CD86}}}.$$

RESULTS

The improved single-molecule fluorescence-based stoichiometric assay utilizes comparisons with well-characterized monomeric and dimeric control proteins, i.e., CD86 and CD28, and cell fixation to limit artifacts arising from differences in the distribution and diffusion of given test proteins versus these controls (Fig. S1). Proteins are transiently expressed in HEK-293T or CHO-K1 cells as chimeras with either SNAP-tags (25) or HaloTags (26) for organic fluorophore labeling at the (intracellular) C-terminus (Fig. 1, *A* and *C*). Transient transfection ensures that the lower surface expression levels (typically 0.1–1 receptors/μm^2) required to minimize random colocalization are achievable, and C-terminal labeling avoids difficulties we have experienced with the folding and trafficking of some N-terminally tagged

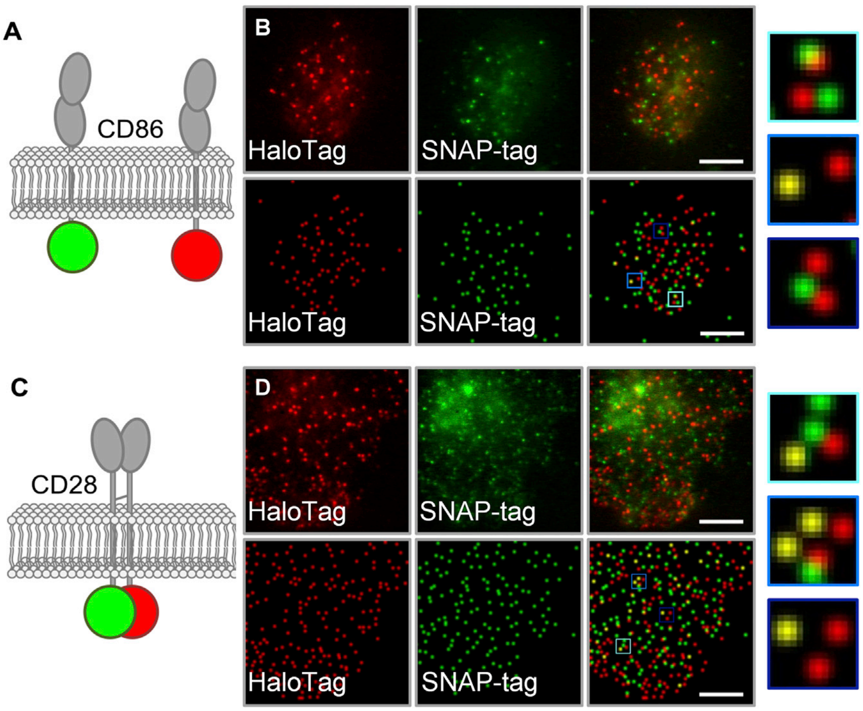

FIGURE 1 Principle of referenced colocalization exemplified for CD86 and CD28 controls expressed in HEK-293T cells. (*A*) Representation of transfected HaloTag- (*red*) and SNAP-tag (*green*)-labeled monomeric CD86 protein. (*B*) Representative data obtained for CD86 in the green and red channels showing raw data (*top row*) and reconstructions of spot detection after application of the tracking algorithm (*bottom row*). Blue-boxed regions are shown magnified at far right. (*C*) HaloTag-(*red*) and SNAP-tag-labeled (*green*) dimeric CD28 protein. (*D*) Representative raw and reconstructed data obtained for CD28 showing higher levels of coincidence. Scale bars are 5 μm.

GPCRs (data not shown). The labeled proteins are imaged in fixed cells for 400 frames with an exposure time of 35 ms, after which individual fluorescent puncta are identified using a custom algorithm (22). Multiple frames are collected to exclude the small fraction of residual mobile proteins that would otherwise bias the measurements of coincidence. Fixation is generally highly effective at reducing the movement of the majority of fluorescent objects to levels comparable with those of glass-immobilized SNAP-tag and HaloTag ligands (Fig. S2). Typically, <6% of spots on fixed cells exhibited movement greater than that required to leave the diffraction-limited starting position within 10 frames. Representative examples of spot detection are given in Figs. 1, *B* and *D*, 2 *B*, and 3 *B*.

In the experiments presented here, the number of SNAP-tag fluorescent puncta was considerably larger than the number of equivalent HaloTag fluorescent puncta, presumably due to differences in folding and labeling efficiency. Cells with a fluorescent SNAP-tag/HaloTag ratio in the range 6–1 were used for data collection, and analysis was limited to cells exhibiting 100–1000 individual HaloTag diffraction-limited single emitters (Fig. S3 and Table 2). Coincidence was calculated relative to the HaloTag channel due to the lower densities of objects in this channel. Coincident events are defined as spatially isolated, diffraction-limited fluorescent HaloTag puncta that are within 300 nm of a fluorescent SNAP-tag spot. The distance criterion is based on the positional accuracy for the red and blue channel alignment, and the use of 10 frames ensures an optimal signal/noise ratio. The results are not sensitive to the distance criterion (Fig. S5) or number of frames (see Materials and Methods) used to identify a coincident event.

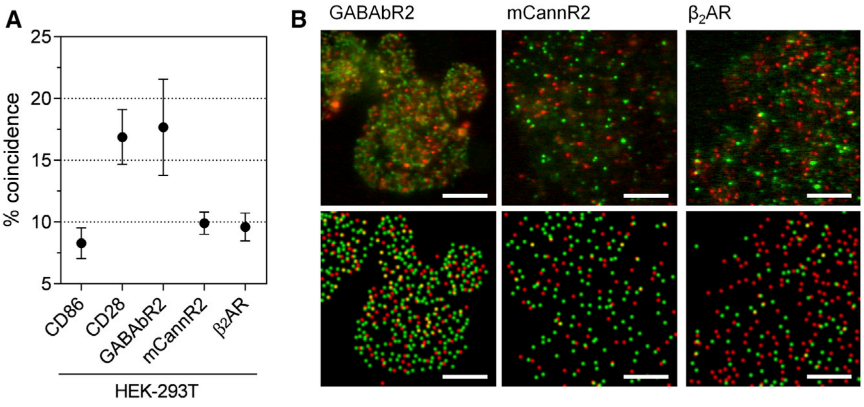

FIGURE 2 Referenced colocalization analysis differentiates between GPCR oligomerization states in HEK-293T cells. (*A*) Coincidence for CD28 and GABAbR2, but not mCannR2 or β_2AR, is significantly larger ($p > 0.05$, two-tailed *t*-test) than that measured for CD86. Values are mean percentage coincidence $\pm$ SE for data from individual cells. (*B*) Representative images showing fluorescence of GPCRs expressed in HEK-293T cells, in the form of both raw data (*top*) and reconstructed data after spot detection (*bottom*). Equivalent images for CD86 and CD28 are given in Fig. 1. Red spots correspond to fluorescent HaloTag-labeled proteins and green spots to fluorescent SNAP-tag-labeled proteins. Scale bars are 5 μm.

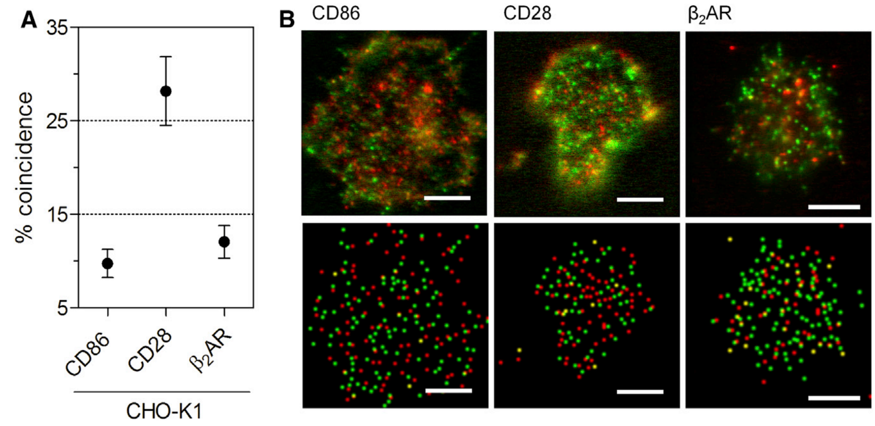

FIGURE 3 Referenced colocalization analysis reveals that β_2AR does not behave as a dimer in CHO-K1 cells. (*A*) The coincidence value for β_2AR is not significantly larger than that measured for CD86. (*B*) Representative raw (*top*) and reconstructed (*bottom*) data obtained for HaloTag- (*red*) and SNAP-tag-labeled (*green*) CD86, CD28, and β_2AR expressed in CHO-K1 cells. Scale bars are 5 μm.

HEK-293T cells were used initially to facilitate comparisons with our previous RET experiments (16,27). We first established the dynamic range of the assay by measuring the coincidence levels for the monomer control CD86 and the dimer CD28 in these cells, and the values were 8.2 ± 1.2% and 16.9 ± 2.2%, respectively (Fig. 2 *A*; values given are the mean ± SE coincidence observed for individual cells within a range of fluorescent SNAP-tag/HaloTag spot ratio of 6:1 and 100–1000 fluorescent HaloTag events/cell). The differences in these coincidence values are more than sufficient to unambiguously distinguish between strict monomers and dimers. Coincidence for CD86 results from fluorescent HaloTag- and SNAP-tag puncta that, by chance, are close enough to be recorded as a coincident event, which depends simply on the density of fluorescent HaloTag- and SNAP-tag puncta. However, if there is nonrandom diffusion on the cell surface, e.g., due to the underlying cytoskeleton, and hence a nonrandom spatial distribution of CD86, the level of coincidence will be increased above that governed by chance (5). We suspected that the reason the coincidence level for CD28 was not higher was due to relatively low labeling efficiencies. To directly measure the SNAP-tag and HaloTag labeling efficiencies, we determined the levels of coincidence for our fluorescently labeled SNAP-tag and HaloTag CD86 constructs bound to fluorescently tagged anti-CD86 antibodies. This showed that the labeling efficiencies were ~33% for the HaloTag and ~16% for the SNAP-tag (Fig. S4). The value of 16% for the SNAP-tag labeling efficiency is comparable to the value of 21.2% calculated from the coincidence level for CD28 (see Materials and Methods). This strongly suggests that it is principally the labeling efficiency that limits the coincidence value for the CD28 dimer control rather than issues to do with spot detection. An important advantage of the improved method, therefore, is that by establishing the dynamic range using control proteins, stoichiometry can be assigned without the need for 1) high labeling efficiencies, which are technically very difficult to obtain, or 2) the need to measure the labeling efficiency or the expression levels of the constructs.

Having established the dynamic range of the assay, we then compared the levels of coincidence obtained for proteins of interest with those measured for the monomer and dimer controls. The mouse *Rhodopsin*-family GPCR, cannabinoid receptor 2 (mCannR2) exhibited a coincidence level not significantly different from that of CD86, i.e., 9.9 ± 0.9% (Fig. 2). In contrast, the *Glutamate*-family GABAbR2, which uses N-terminal domains to homodimerize (2,16), exhibited a coincidence level comparable to that of CD28, i.e., 17.7 ± 3.9%, as expected (Fig. 2; the *p*-values

TABLE 2 Number of analyzed cells and fluorescent spots, and statistical probability of a difference from CD86 in the same cell line, for each candidate protein

Protein	Cell Line	Cells Imaged	Noncoincident HaloTag Spots	Coincident HaloTag Spots	% Coincidence ± SE	*p*-Value of Difference from CD86
CD86	HEK-293T	13	4279	346	8.2 ± 1.2	–
CD28	HEK-293T	7	2952	587	16.9 ± 2.2	0.001
GABAbR2	HEK-293T	7	2844	622	17.7 ± 3.9	0.012
mCannR2	HEK-293T	16	9166	978	9.9 ± 0.9	0.289
β_2AR	HEK-293T	13	8132	855	9.6 ± 1.1	0.440
CD86	CHO-K1	10	2797	313	9.7 ± 1.5	–
CD28	CHO-K1	8	1626	678	28.2 ± 3.7	0.0001
β_2AR	CHO-K1	10	2497	334	12.1 ± 1.7	0.325

Statistical probability of a difference was determined using a two-tailed *t* test. Coincidence values were determined as the mean coincidence for each cell imaged.

obtained for comparisons with CD86 are given in Table 2). Analysis of the same data using different distance criteria to define coincidence did not affect the assignment of mCannR2 or GABAbR2 stoichiometry on the basis of comparisons with CD86 or CD28 (Fig. S5 *A*). These data argue against mCannR2 homodimerization, and they confirm that the assay is capable of correctly identifying GPCR dimers.

We then extended the analysis to the prototypical *Rhodopsin* family GPCR, β_2AR, and this gave a coincidence level of 9.6 ± 1.1 in HEK-293T cells (Fig. 2), i.e., a level that was very similar to that of mCannR2 and not significantly different from that of the monomer control CD86. This suggests that β_2AR behaves as a monomer, consistent with our previous RET-based analyses of this receptor in these cells (13,24). An important caveat, however, is that HEK-293T cells express low levels of native (unlabeled) β_2AR (28), which could have reduced the coincidence signal due to competition effects.

To avoid this possibility, we performed the stoichiometric analysis of β_2AR again, this time in CHO-K1 cells, as these cells do not have native human β_2AR and do not cluster the receptor (2,29), so that misidentification of clustered monomers as dimers is less likely. This required the dynamic range for these cells to be established, wherein CD86 and CD28 yielded coincidence values of 9.7 ± 1.5% and 28.2 ± 3.7% (Fig. 3 *D*). As expected, the level of coincidence for CD86 is unchanged versus that in HEK-293T cells. Because the level and range of fluorescent HaloTag and SNAP-tag spots to be used in the analyses have been defined on a per-cell basis, the chance level of CD86 coincidence will not vary from one cell type to another irrespective of expression level (the relationship between expression level and labeling efficiency for monomers and dimers is illustrated schematically in Fig. S6). The coincidence value for CD28, however, is substantially higher than that obtained in HEK-293T cells. This is readily explained by the SNAP-tag labeling efficiency being higher in CHO-K1 cells, since 1) the coincidence level for dimers will be sensitive to labeling efficiency and expression level (Fig. S6), and 2) labeling efficiency is known to vary between different cell types (see, e.g., Klein et al. (30)). Using the coincidence value for CD28, we calculate that the SNAP-tag and HaloTag labeling efficiencies were 31% and 62% (the calculations are given in Materials and Methods and the Supporting Discussion). These considerations once again emphasize the importance of determining the dynamic range of the assay using well-defined control proteins. The coincidence value obtained for β_2AR in CHO-K1 cells was not significantly higher than for CD86 (12.1 ± 1.7%; Fig. 3), consistent once again with β_2AR behaving as a monomer. Changing the distance criterion used to define coincidence did not affect the conclusions drawn on the basis of the CHO-K1 measurements (Fig. S5 *B*).

Using the measured mean ± SE values, it is possible to estimate the degree of receptor dimerization that would have been required to achieve significance in our assay (see Materials and Methods). The level of dimerization of mCannR2 required for 90% confidence is 29% ($t = 1.703$, 27 degrees of freedom), and for β_2AR, it is 32% in HEK-293T cells ($t = 1.717$, 22 degrees of freedom) and 21% in CHO-K1 cells ($t = 1.734$, 18 degrees of freedom). We can therefore conclude that the dimerization of mCannR2 and β_2AR, if it occurs at all, cannot exceed these levels in our assay. Indeed, for β_2AR, even 16% dimerization would still have been detectable with 80% confidence ($t = 1.33$, 18 degrees of freedom), but this was not observed.

DISCUSSION

We have demonstrated the utility of an improved single-molecule imaging-based stoichiometric assay that is reliant on comparisons with well-characterized control proteins and incorporates cell fixation to limit artifacts arising from variation in protein distribution and diffusion. This means that we do not need to know the relative expression level of the SNAP-tag and HaloTag constructs or their labeling efficiencies. We demonstrate the ability of the assay to reliably distinguish between GPCRs shown previously to behave as dimers and monomers, and then use it to address the stoichiometry of a more contentious receptor, β_2AR.

A fundamental problem with the use of single-molecule fluorescence-based methods to determine the oligomerization state of proteins is that, generally, most methods used to label the proteins with fluorophores are not 100% efficient. This can be attributed to inefficient chemical reactions in the case of SNAP-tag- or HaloTag-based labeling, or to the failure of the protein to fold correctly into a fluorescent state in the case of autofluorescent proteins (31). To address this issue, new methods need to be found that increase the labeling efficiency to close to 100% if possible, or else the labeling efficiency and relative expression levels need to be measured and taken into account in the analysis. However, it is generally quite difficult to measure labeling efficiency and relative expression level. A key advantage over older methods, to our knowledge, is that the new approach does not require such measurements because the level of coincidence of the protein of interest is directly compared with the level of coincidence for well-characterized monomer and dimer controls expressed in the same cell line. Although we have used a two-color coincidence approach to analyze our single-molecule data here, the same approach based on monomer and dimer controls could in principle be applied to intensity-based analyses of the data. The method presented here therefore comprises, to our knowledge, a new general approach for more accurately determining whether proteins exist as monomers or dimers (and higher-order oligomers) in the resting cell membrane. The assay could in principle also be used to determine whether receptors alter their stoichiometry after activation. However, additional technical issues arising from, e.g., internalization of

the receptor and the mode and timing of fixation would need to be resolved to perform such experiments.

Our observations are consistent with β_2AR, like mCannR2, behaving as a monomer, in agreement with our previous studies of these receptors using RET-based approaches (16,27), although we are unable to fully exclude the possibility that small fractions of these receptors form dimers. Although this provides only a partial insight into the general behavior of *Rhodopsin*-family receptors, there is at present no evidence suggesting that either β_2AR or mCannR2 are anomalous versus the behavior of *Rhodopsin* family GPCRs generally. Although the stoichiometry of mCannR2 has not been exhaustively studied, β_2AR has been the subject of numerous, often contradictory investigations. Some studies have shown that β_2AR self-associates upon solubilization (32–34), whereas feasible homodimers are conspicuously absent in the lattices of all the published β_2AR crystal structures (35–37). Moreover, isolated β_2AR receptors can efficiently activate G-proteins (13,38), and the receptor crystallizes in a stable 1:1 receptor/G-protein complex (39). In marked contrast, early in situ analyses utilizing RET were claimed to indicate the existence of constitutive β_2AR dimers (32,34,40,41), although the interpretation of much of these data did appear to be problematic (15,16,27), and subsequent RET approaches performed by ourselves and others (42) have instead suggested that the β_2AR is monomeric. Similarly, assays based on the cointernalization or coclustering of β_2AR monomers also fail to support the notion that this receptor oligomerizes (43,44).

Our improved single-molecule imaging-based assay offers yet more support for β_2AR behaving exclusively or almost exclusively as a monomer. A previous single-molecule-level investigation of β_2AR (2) reported high levels of receptor dimerization, and the reasons for this discrepancy are unclear, as the experimental approaches used are very different. Important differences include the use of 1) intensity analysis versus two-color coincidence, 2) live versus fixed cells, 3) stable versus transient expression, and 4) extracellular N-terminal labeling versus cytoplasmic C-terminal labeling. The N-terminal regions of *Rhodopsin*-family GPCRs undergo extensive interactions with extracellular loops (45) and in some cases form disulfide bridges that stabilize the loop orientation (46), implicating the N-terminal regions in receptor folding. We have experienced difficulties expressing N-terminally tagged GPCRs and envisage that N-terminal labeling could result in partial receptor misfolding and aggregation, influencing apparent stoichiometry. In contrast, GPCR C-terminal domains are more typically unstructured (47,48), and C-terminal tagging of receptors is the general approach adopted by the field (49). Our finding, i.e., that the β_2AR does not appreciably dimerize when C-terminally tagged, is consistent with previous single-molecule studies of GPCRs showing that these receptors exhibit primarily monomeric behavior (3,6,19). These observations suggest that at least some *Rhodopsin* family GPCRs are likely to be functionally monomeric, and our improved assay will allow us to determine if this is true for most of these receptors.

SUPPORTING MATERIAL

Supporting Discussion and six figures are available at http://www.biophysj.org/biophysj/supplemental/S0006-3495(15)00934-0.

AUTHOR CONTRIBUTIONS

S.L.L., J.H.F., S.J.D., and D.K. designed the research. S.L.L. and J.H.F. performed the research. L.W. contributed analytical tools. S.L.L., J.H.F., and S.F.L. analyzed the data. S.L.L., J.H.F., S.F.L., S.J.D., and D.K. wrote the article.

ACKNOWLEDGMENTS

This work was supported by the Wellcome Trust and the United Kingdom Medical Research Council. We would like to thank the Royal Society for the University Research Fellowship of SFL (UF120277).

REFERENCES

1. Leake, M. C., J. H. Chandler, …, J. P. Armitage. 2006. Stoichiometry and turnover in single, functioning membrane protein complexes. *Nature.* 443:355–358.
2. Calebiro, D., F. Rieken, …, M. J. Lohse. 2013. Single-molecule analysis of fluorescently labeled G-protein-coupled receptors reveals complexes with distinct dynamics and organization. *Proc. Natl. Acad. Sci. USA.* 110:743–748.
3. Kasai, R. S., K. G. N. Suzuki, …, A. Kusumi. 2011. Full characterization of GPCR monomer-dimer dynamic equilibrium by single molecule imaging. *J. Cell Biol.* 192:463–480.
4. Kohout, S. C., M. H. Ulbrich, …, E. Y. Isacoff. 2008. Subunit organization and functional transitions in Ci-VSP. *Nat. Struct. Mol. Biol.* 15:106–108.
5. Moertelmaier, M., M. Brameshuber, …, H. Stockinger. 2005. Thinning out clusters while conserving stoichiometry of labeling. *Appl. Phys. Lett.* 87:263903.
6. Hern, J. A., A. H. Baig, …, N. J. M. Birdsall. 2010. Formation and dissociation of M1 muscarinic receptor dimers seen by total internal reflection fluorescence imaging of single molecules. *Proc. Natl. Acad. Sci. USA.* 107:2693–2698.
7. Dunne, P. D., R. A. Fernandes, …, D. Klenerman. 2009. DySCo: quantitating associations of membrane proteins using two-color single-molecule tracking. *Biophys. J.* 97:L5–L7.
8. Daumas, F., N. Destainville, …, L. Salomé. 2003. Confined diffusion without fences of a G-protein-coupled receptor as revealed by single particle tracking. *Biophys. J.* 84:356–366.
9. Vobornik, D., Y. Rouleau, …, J. P. Pezacki. 2009. Nanoscale organization of β_2-adrenergic receptor-Venus fusion protein domains on the surface of mammalian cells. *Biochem. Biophys. Res. Commun.* 382:85–90.
10. Gurevich, V. V., and E. V. Gurevich. 2008. How and why do GPCRs dimerize? *Trends Pharmacol. Sci.* 29:234–240.
11. Kasai, R. S., and A. Kusumi. 2014. Single-molecule imaging revealed dynamic GPCR dimerization. *Curr. Opin. Cell Biol.* 27:78–86.
12. Bayburt, T. H., A. J. Leitz, …, S. G. Sligar. 2007. Transducin activation by nanoscale lipid bilayers containing one and two rhodopsins. *J. Biol. Chem.* 282:14875–14881.

13. Whorton, M. R., M. P. Bokoch, …, R. K. Sunahara. 2007. A monomeric G protein-coupled receptor isolated in a high-density lipoprotein particle efficiently activates its G protein. *Proc. Natl. Acad. Sci. USA.* 104:7682–7687.

14. Kuszak, A. J., S. Pitchiaya, …, R. K. Sunahara. 2009. Purification and functional reconstitution of monomeric μ-opioid receptors: allosteric modulation of agonist binding by Gi2. *J. Biol. Chem.* 284:26732–26741.

15. Felce, J. H., and S. J. Davis. 2012. Unraveling receptor stoichiometry using BRET. *Front. Endocrinol.* (*Lausanne*). 3:86.

16. James, J. R., M. I. Oliveira, …, S. J. Davis. 2006. A rigorous experimental framework for detecting protein oligomerization using bioluminescence resonance energy transfer. *Nat. Methods.* 3:1001–1006.

17. Chabre, M., P. Deterre, and B. Antonny. 2009. The apparent cooperativity of some GPCRs does not necessarily imply dimerization. *Trends Pharmacol. Sci.* 30:182–187.

18. Chabre, M., and M. le Maire. 2005. Monomeric G-protein-coupled receptor as a functional unit. *Biochemistry.* 44:9395–9403.

19. Nenasheva, T. A., M. Neary, …, J. E. Molloy. 2013. Abundance, distribution, mobility and oligomeric state of M_2 muscarinic acetylcholine receptors in live cardiac muscle. *J. Mol. Cell. Cardiol.* 57:129–136.

20. Lambert, N. A. 2010. GPCR dimers fall apart. *Sci. Signal.* 3:pe12.

21. Linsley, P. S., S. G. Nadler, …, S.-Y. Shaw. 1995. Binding stoichiometry of the cytotoxic T lymphocyte-associated molecule-4 (CTLA-4). A disulfide-linked homodimer binds two CD86 molecules. *J. Biol. Chem.* 270:15417–15424.

22. Weimann, L., K. A. Ganzinger, …, D. Klenerman. 2013. A quantitative comparison of single-dye tracking analysis tools using Monte Carlo simulations. *PLoS One.* 8:e64287.

23. Tanaka, K. A. K., K. G. N. Suzuki, …, A. Kusumi. 2010. Membrane molecules mobile even after chemical fixation. *Nat. Methods.* 7:865–866.

24. Koyama-Honda, I., K. Ritchie, …, A. Kusumi. 2005. Fluorescence imaging for monitoring the colocalization of two single molecules in living cells. *Biophys. J.* 88:2126–2136.

25. Keppler, A., S. Gendreizig, …, K. Johnsson. 2003. A general method for the covalent labeling of fusion proteins with small molecules in vivo. *Nat. Biotechnol.* 21:86–89.

26. Los, G. V., L. P. Encell, …, K. V. Wood. 2008. HaloTag: a novel protein labeling technology for cell imaging and protein analysis. *ACS Chem. Biol.* 3:373–382.

27. Felce, J. H., R. G. Knox, and S. J. Davis. 2014. Type-3 BRET, an improved competition-based bioluminescence resonance energy transfer assay. *Biophys. J.* 106:L41–L43.

28. Ménard, L., S. S. G. Ferguson, …, L. S. Barak. 1997. Synergistic regulation of β_2-adrenergic receptor sequestration: intracellular complement of β-adrenergic receptor kinase and β-arrestin determine kinetics of internalization. *Mol. Pharmacol.* 51:800–808.

29. Scarselli, M., P. Annibale, and A. Radenovic. 2012. Cell type-specific β_2-adrenergic receptor clusters identified using photoactivated localization microscopy are not lipid raft related, but depend on actin cytoskeleton integrity. *J. Biol. Chem.* 287:16768–16780.

30. Klein, T., A. Löschberger, …, M. Sauer. 2011. Live-cell dSTORM with SNAP-tag fusion proteins. *Nat. Methods.* 8:7–9.

31. Jackson, S. E., T. D. Craggs, and J. R. Huang. 2006. Understanding the folding of GFP using biophysical techniques. *Expert Rev. Proteomics.* 3:545–559.

32. Angers, S., A. Salahpour, …, M. Bouvier. 2000. Detection of β_2-adrenergic receptor dimerization in living cells using bioluminescence resonance energy transfer (BRET). *Proc. Natl. Acad. Sci. USA.* 97:3684–3689.

33. Cao, T. T., A. Brelot, and M. von Zastrow. 2005. The composition of the β-2 adrenergic receptor oligomer affects its membrane trafficking after ligand-induced endocytosis. *Mol. Pharmacol.* 67:288–297.

34. Ramsay, D., E. Kellett, …, G. Milligan. 2002. Homo- and hetero-oligomeric interactions between G-protein-coupled receptors in living cells monitored by two variants of bioluminescence resonance energy transfer (BRET): hetero-oligomers between receptor subtypes form more efficiently than between less closely related sequences. *Biochem. J.* 365:429–440.

35. Cherezov, V., D. M. Rosenbaum, …, R. C. Stevens. 2007. High-resolution crystal structure of an engineered human β_2-adrenergic G protein-coupled receptor. *Science.* 318:1258–1265.

36. Hanson, M. A., V. Cherezov, …, R. C. Stevens. 2008. A specific cholesterol binding site is established by the 2.8 Å structure of the human β_2-adrenergic receptor. *Structure.* 16:897–905.

37. Rasmussen, S. G. F., H.-J. Choi, …, B. K. Kobilka. 2007. Crystal structure of the human β_2 adrenergic G-protein-coupled receptor. *Nature.* 450:383–387.

38. Leitz, A. J., T. H. Bayburt, …, S. G. Sligar. 2006. Functional reconstitution of β_2-adrenergic receptors utilizing self-assembling Nanodisc technology. *Biotechniques.* 40:601–602, 604, 606 passim.

39. Rasmussen, S. G. F., B. T. DeVree, …, B. K. Kobilka. 2011. Crystal structure of the β2 adrenergic receptor-Gs protein complex. *Nature.* 477:549–555.

40. Breit, A., M. Lagacé, and M. Bouvier. 2004. Hetero-oligomerization between β_2- and β_3-adrenergic receptors generates a β-adrenergic signaling unit with distinct functional properties. *J. Biol. Chem.* 279:28756–28765.

41. Lavoie, C., J. F. Mercier, …, T. E. Hébert. 2002. β_1/β_2-adrenergic receptor heterodimerization regulates β_2-adrenergic receptor internalization and ERK signaling efficacy. *J. Biol. Chem.* 277:35402–35410.

42. Kawano, K., Y. Yano, …, K. Matsuzaki. 2013. Stoichiometric analysis of oligomerization of membrane proteins on living cells using coiled-coil labeling and spectral imaging. *Anal. Chem.* 85:3454–3461.

43. Lan, T.-H., S. Kuravi, and N. A. Lambert. 2011. Internalization dissociates β_2-adrenergic receptors. *PLoS One.* 6:e17361.

44. Gavalas, A., T.-H. Lan, …, N. A. Lambert. 2013. Segregation of family A GPCR protomers in the plasma membrane. *Mol. Pharmacol.* 84:346–352.

45. Venkatakrishnan, A. J., X. Deupi, …, M. M. Babu. 2013. Molecular signatures of G-protein-coupled receptors. *Nature.* 494:185–194.

46. Wu, B., E. Y. T. Chien, …, R. C. Stevens. 2010. Structures of the CXCR4 chemokine GPCR with small-molecule and cyclic peptide antagonists. *Science.* 330:1066–1071.

47. Unal, H., and S. S. Karnik. 2012. Domain coupling in GPCRs: the engine for induced conformational changes. *Trends Pharmacol. Sci.* 33:79–88.

48. Jaakola, V. P., J. Prilusky, …, A. Goldman. 2005. G protein-coupled receptors show unusual patterns of intrinsic unfolding. *Protein Eng. Des. Sel.* 18:103–110.

49. Pfleger, K. D. G., and K. A. Eidne. 2005. Monitoring the formation of dynamic G-protein-coupled receptor-protein complexes in living cells. *Biochem. J.* 385:625–637.

SUPPORTING MATERIAL

Referenced single-molecule measurements differentiate between GPCR oligomerization states

Sarah L. Latty,*[§] James H. Felce,[† §] Laura Weimann, * Steven F. Lee,* Simon J. Davis,[† ¶] and David Klenerman *[¶]

*Department of Chemistry, University of Cambridge, Lensfield Road, Cambridge CB2 1EW, United Kingdom; [†]Radcliffe Department of Clinical Medicine and Medical Research Council Human Immunology Unit, Weatherall Institute of Molecular Medicine, University of Oxford, Oxford OX3 9DS, United Kingdom. [§] These authors contributed equally. [¶] Corresponding authors.

SUPPORTING RESULTS

- **FIGURE S1 Diffusional behavior affects apparent stoichiometry**
- **FIGURE S2 Histograms of room temperature diffusion coefficients of fixed and unfixed puncta**
- **FIGURE S3 Expression levels of HaloTag constructs in HEK-293T and CHO-K1 cells**
- **FIGURE S4 Estimation of SNAP-tag/HaloTag labeling efficiency using antibody-staining**
- **FIGURE S5 Coincidence values obtained with a varying distance criterion**
- **FIGURE S6 The dependence of coincidence on labeling efficiency for monomers and dimers**

SUPPORTING DISCUSSION

SUPPORTING REFERENCES

SUPPORTING RESULTS

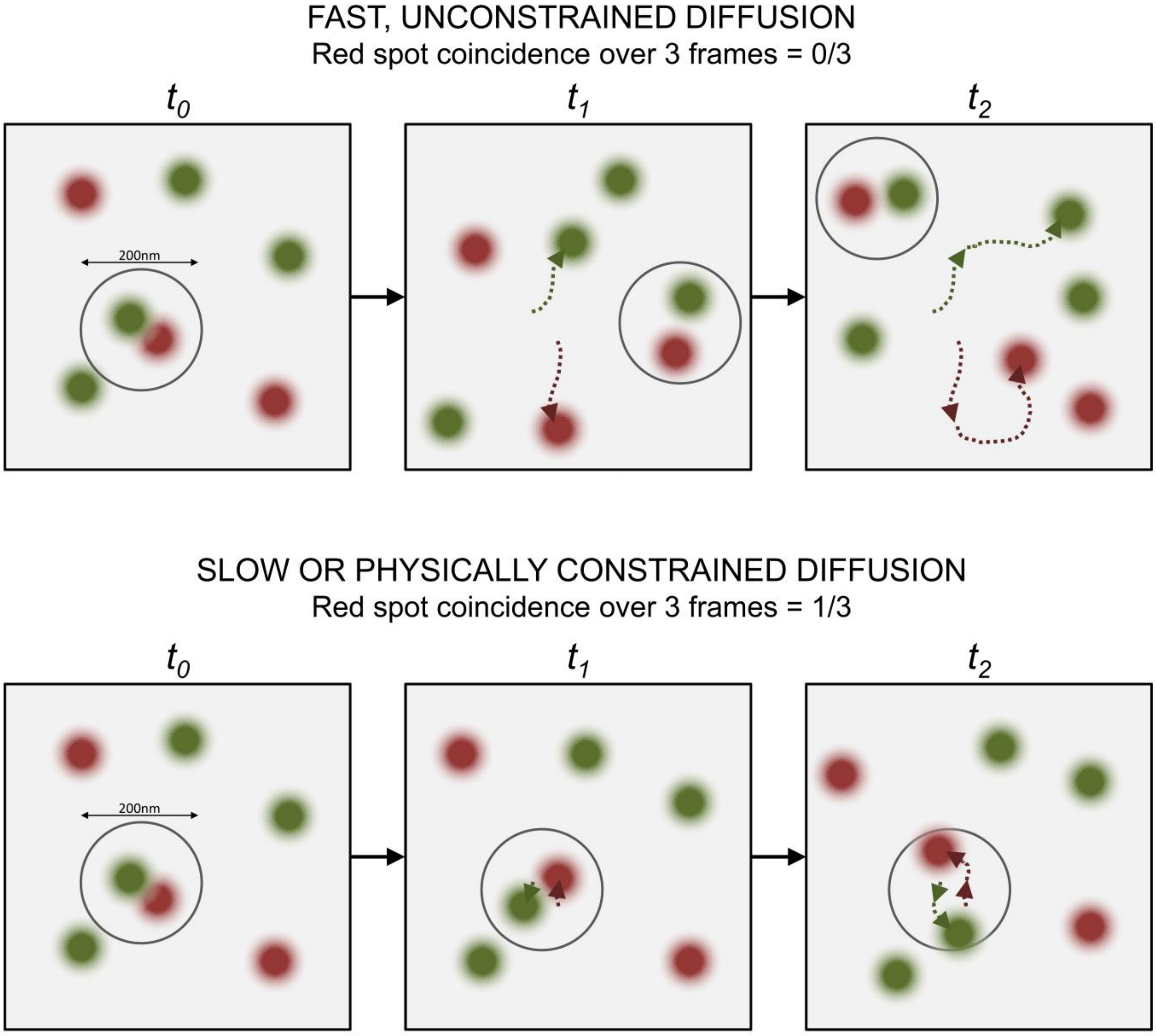

FIGURE S1 Diffusional behavior affects apparent stoichiometry: proteins that have equivalent global distributions within a membrane can have different levels of coincidence if they have different diffusional behavior. Red and green spots represent proteins labeled in two colors. Circles indicate the resolution of diffraction-limited microscopy (typically 200-300nm) within which two spots will be colocalized. Diffusional paths for the starting colocalized pair are shown as broken arrows. For fast diffusing proteins (top row), the number of spots within the colocalization distance of a spot of the opposite color will remain constant at any given time-point (t_0, t_1, t_2, *etc.*). However, for a spot to be identified as coincident, it must remain colocalized with the same partner for a defined number of frames. Although there will be colocalized spots at any given time-point, very few will remain colocalized for sufficient time to be identified as coincident. Hence, coincidence will be low for rapidly diffusing proteins. Proteins that diffuse more slowly or are physically constrained in their diffusion (bottom row) are more likely to remain within the colocalization distance of the same partner for multiple frames, and so coincidence will be higher than for more rapidly diffusing proteins at the same density. Fixation removes the variable of diffusion, and all proteins with the same stoichiometry and density will exhibit the same level of coincidence. This allows comparison to controls of known stoichiometry without the risk of diffusion inadvertently affecting the comparisons.

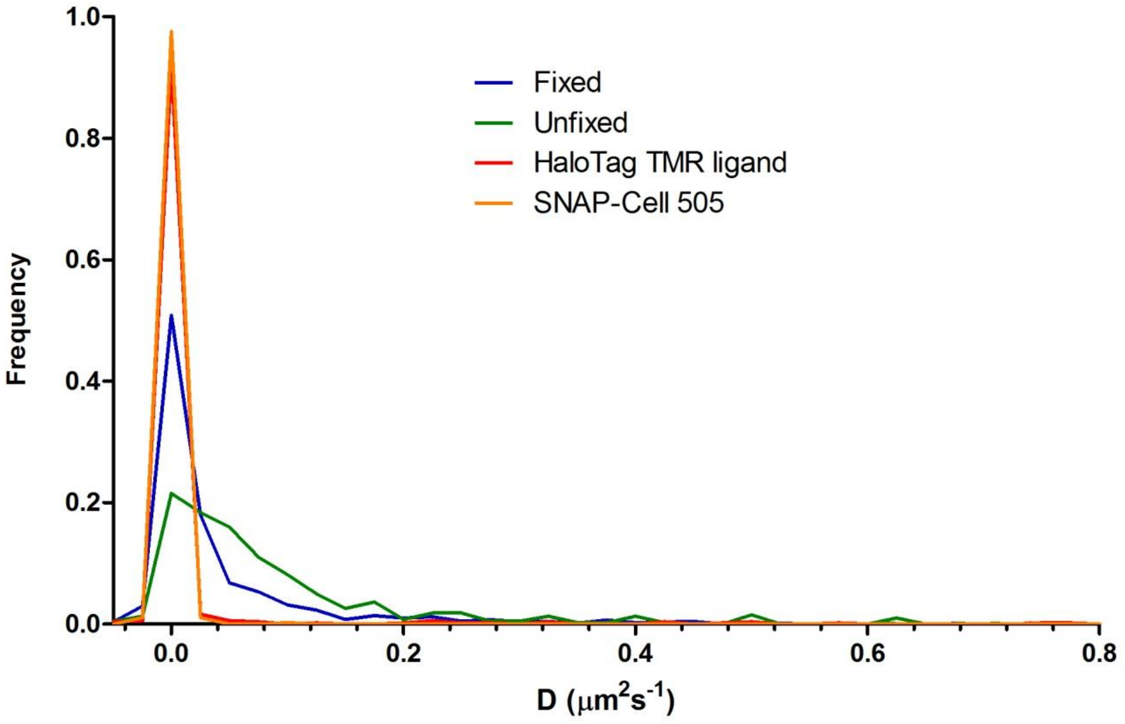

FIGURE S2 Histograms of diffusion coefficients of tagged CD86 on unfixed and fixed cells. Diffusional data for TMR and SNAP-Cell 505 fluorophores adsorbed to glass is also displayed. Diffusion coefficients were calculated using a Mean-Square Displacement (MSD) analysis. Assuming 'random walk' receptor motion a linear function was utilized to relate MSD to Brownian diffusion (1). Apparently negative diffusion coefficients should be interpreted as having a diffusion coefficient of zero within experimental error. Such coefficients arise by chance for some molecules undergoing very small motions as MSD plots can produce a negative slope i.e. the molecule moves further at short times than long times. Across all collected puncta for all fixed proteins, 5.9% had a diffusion coefficient greater than $0.2 \mu m^2 s^{-1}$, which is the limit above which spots will have moved from their original 300nm-diameter area ($0.07 \mu m^2$) within the 10 frame (350ms) period. In unfixed cells, 11.5% of CD86 trajectories had a diffusion coefficient greater than $0.2 \mu m^2 s^{-1}$. The difference in diffusion between fixed and unfixed samples was tested using a Kolmogorov-Smirnov comparison and found to be significant to $p<0.001$ with a maximum difference of cumulative distributions (D) of 0.37. Fixed trajectories had a reduced difference in diffusion relative to adsorbed fluorophores (D=0.55) compared to unfixed puncta (D=0.80). The fixation strategy, therefore, is effective in immobilizing the majority of tagged proteins.

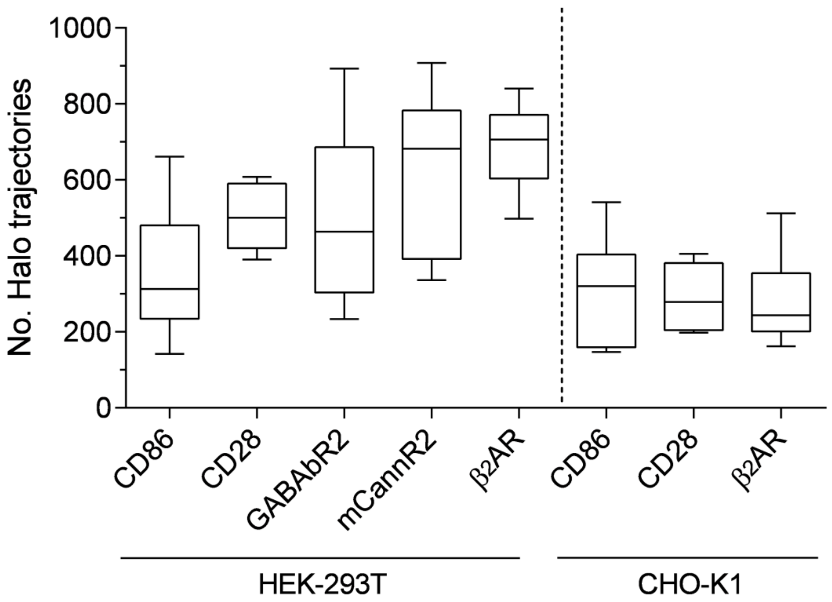

FIGURE S3 Expression levels of HaloTag constructs in HEK-293T and CHO-K1 cells. Data are shown as minimum to maximum box-and-whisker plots.

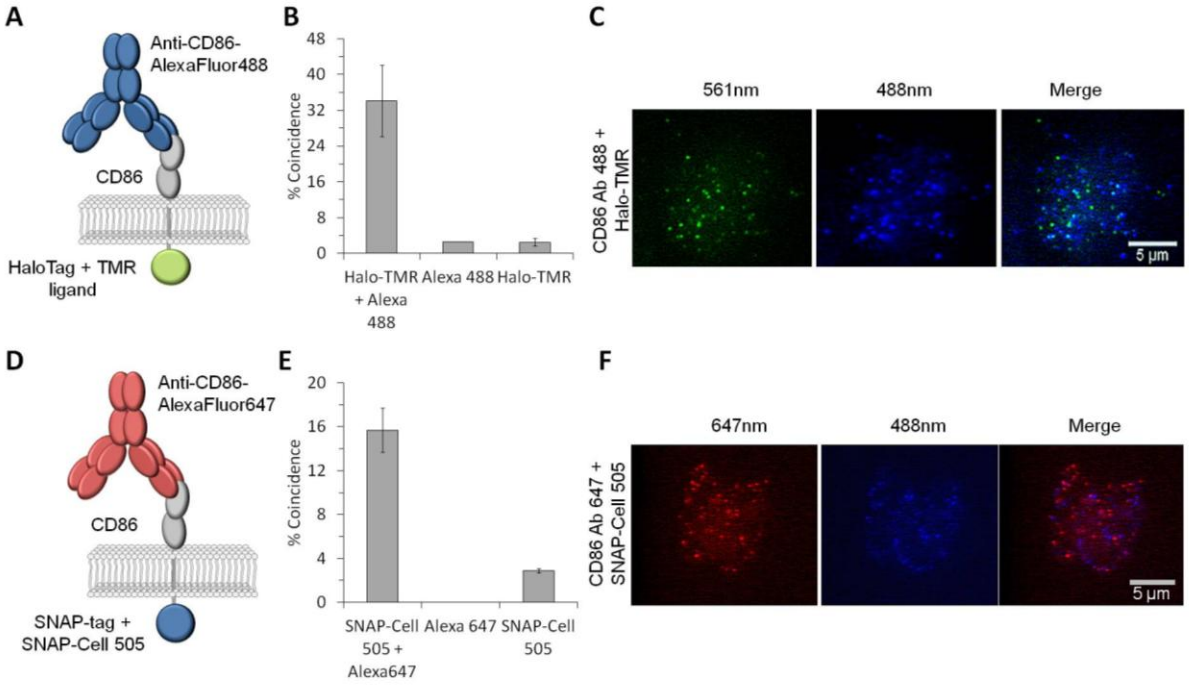

FIGURE S4 Estimation of SNAP-tag/HaloTag labeling efficiency in HEK-293T cells using antibody-staining. (**A** & **D**) Representations of the strategy used in each case: anti-CD86 antibody conjugated to either AlexaFluor 647 or AlexaFluor 488 was used to provide a second label for CD86 fused to either HaloTag or SNAP-tag. Antibodies were used at 100 µg/ml to ensure saturating, monovalent binding. (**B** & **E**) Coincidence values for spots in the two relevant colors. Coincidence is given relative to antibody spots, i.e. coincidence represents the percentage of antibody spots for which an associated tag ligand spot was detected. Images were collected on cells labeled with both antibody and tag ligand, antibody only, and tag ligand only. Bars are mean ± SEM. (**C** & **F**) Representative examples of cells labeled with both antibody and tag ligand.

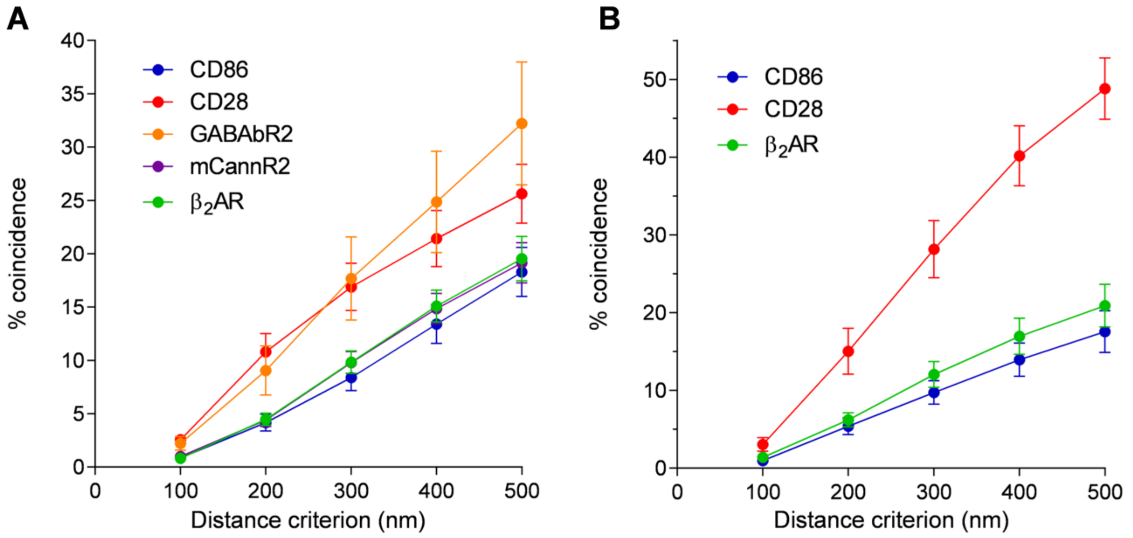

FIGURE S5 Coincidence values obtained with a varying distance criterion. The overall assignment of stoichiometry for GPCRs relative to CD86 and CD28 controls is unaffected by the distance criterion used to define coincidence in both HEK-293T (**A**) and CHO-K1 (**B**) cells. Bars are mean ± SEM.

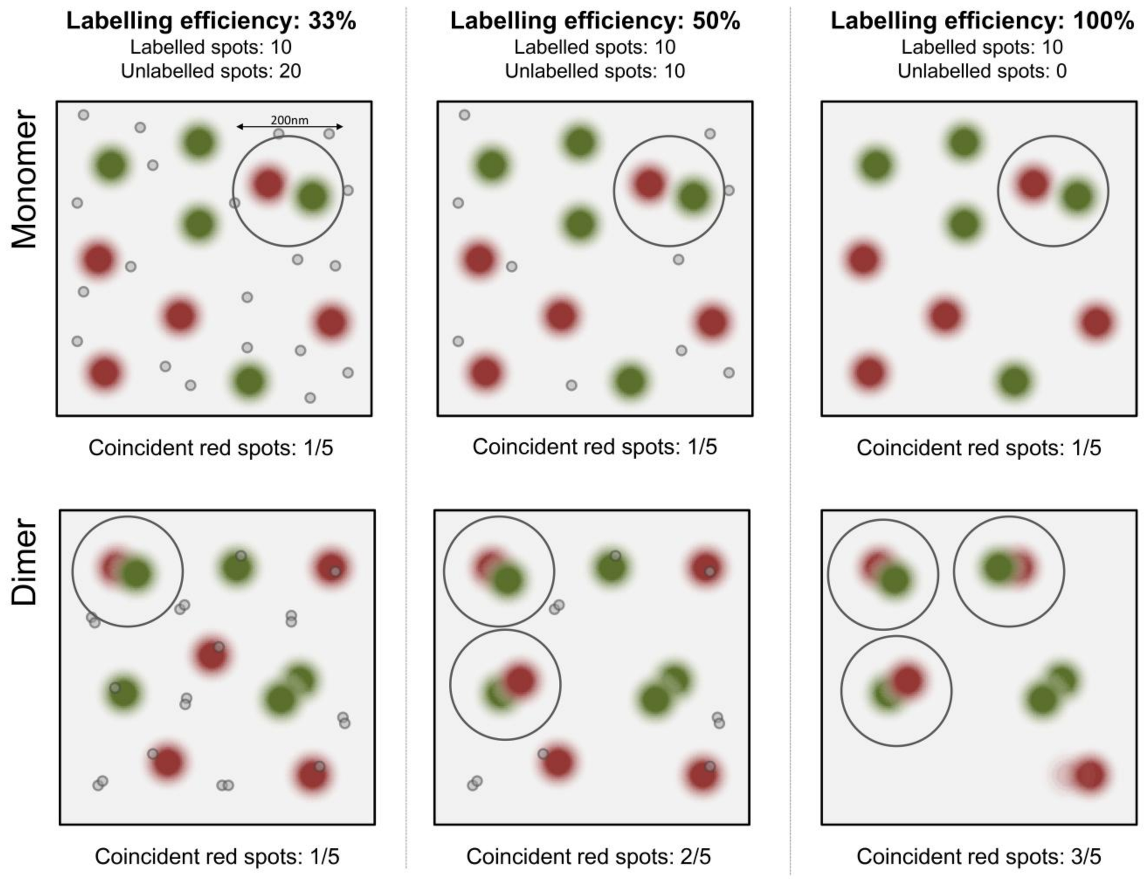

FIGURE S6 The dependence of coincidence on labeling efficiency for monomers and dimers. Proteins are shown as a schematic of labeled (red and green) and unlabelled (grey) spots at varying labeling efficiencies. Circles indicate the resolution of diffraction-limited microscopy (typically 200-300nm) within which two spots will be colocalized. If the total density of labeled proteins is constant, the coincidence of monomeric proteins is independent of labeling efficiency (top row). This is because coincidence of monomers is the result only of random colocalization of labeled proteins within the diffraction limit of the detection method. If labeling efficiency increases but the density of labeled spots remains constant, there will be fewer unlabeled proteins but the coincidence of the labeled proteins will not change. In contrast, the coincidence of dimers is dependent on labeling efficiency as unlabeled proteins compete for position within dimers and hence reduce coincidence. Thus, as labeling efficiency increases, coincidence of dimers also increases (bottom row).

SUPPORTING DISCUSSION

Derivation of the relationship between labeling efficiency, relative expression level, and coincidence.

Let:
n = Fraction all CD28 protomers SNAP-tagged (labeled and unlabeled)
m = Fraction all CD28 protomers Halo-tagged (labeled and unlabeled)
p = SNAP-tag labeling efficiency
q = HaloTag labeling efficiency
f = Ratio of SNAP(labeled):Halo(labeled)
c = Fraction of CD28 dimers containing both HaloTag(labeled) and SNAP-tag(labeled) (*i.e.* coincident fraction)

$\therefore$
$Fraction\ CD28\ protomers\ with\ Halo(labeled) = mq$
$Fraction\ CD28\ protomers\ with\ Halo(unlabeled) = m(1-q)$
$Fraction\ CD28\ protomers\ with\ SNAP(labeled) = np$
$Fraction\ CD28\ protomers\ with\ SNAP(unlabeled) = n(1-p)$

$\therefore$
$Fraction\ dimers\ with\ 2 \times Halo(labeled) = m^2q^2$
$Fraction\ dimers\ with\ 1 \times Halo(labeled) +\ 1 \times SNAP(labeled) = 2mqnp$
$Fraction\ dimers\ with\ 1 \times Halo(labeled) +\ 1 \times SNAP(unlabeled) = 2mqn(1-p)$
$Fraction\ dimers\ with\ 1 \times Halo(labeled) +\ 1 \times Halo(unlabeled) = 2mqm(1-q)$

$$c = \frac{Fraction\ dimers\ with\ 1 \times Halo(labelled) +\ 1 \times SNAP(labelled)}{Fraction\ dimers\ with\ at\ least\ 1\ \times Halo(labelled)}$$

$$= \frac{2mqnp}{(m^2q^2) + (2mqnp) + \left(2mqn(1-p)\right) + \left(2mqm(1-q)\right)}$$

$$= \frac{2np}{2n + 2m - mq}$$

$Since\ n = 1 - m$

$$c = \frac{2p(1-m)}{2 - mq}$$

$\therefore if\ p = q$

$$p = \frac{2c}{2 + cm - 2m}$$

$\therefore if\ 2p = q$

$$p = \frac{c}{1 + cm - m}$$

$$Ratio\ SNAP(labeled){:}\ Halo(labeled) = \frac{Fraction\ CD28\ protomers\ with\ SNAP(labeled)}{Fraction\ CD28\ protomers\ with\ Halo(labeled)}$$

$$\therefore f = \frac{np}{mq} = \frac{np}{(1-n)q} = \frac{np}{q-nq}$$

$$\therefore n = \frac{qf}{qf+p}$$

$$\therefore m = 1 - \left(\frac{qf}{qf+p}\right)$$

$$\therefore if\ p = q$$

$$m = 1 - \left(\frac{f}{(1+f)}\right)$$

$$\therefore if\ 2p = q$$

$$m = 1 - \left(\frac{2f}{(1+2f)}\right)$$

For CD28 in HEK-293T cells

c = observed coincidence = 0.17

f = observed SNAP:Halo ratio = 3.23

$\therefore if\ 2p = q$ $\boldsymbol{m = 0.134}$ $\boldsymbol{p = 19.1\%}$ $\boldsymbol{q = 38.3\%}$

$\therefore if\ p = q$ $\boldsymbol{m = 0.236}$ $\boldsymbol{p = 21.7\%}$ $\boldsymbol{q = 21.7\%}$

For CD28 in CHO-K1 cells

c = observed coincidence = 0.28

f = observed SNAP:Halo ratio = 3.27

$\therefore if\ 2p = q$ $\boldsymbol{m = 0.133}$ $\boldsymbol{p = 30.9\%}$ $\boldsymbol{q = 61.9\%}$

$\therefore if\ p = q$ $\boldsymbol{m = 0.234}$ $\boldsymbol{p = 36.3\%}$ $\boldsymbol{q = 36.3\%}$

For CD28 in HEK-293T cells using measured labeling efficiencies

p = measured SNAP labeling efficiency = 0.16

q = measured Halo labeling efficiency = 0.33

f = observed SNAP:Halo ratio = 3.23

$\therefore m = 0.1305$

$c = \frac{2p(1-m)}{2-mq} = \frac{2\times 0.16\times(1-0.1305)}{2-(0.1305\times 0.33)} = \boldsymbol{0.142}$ **= 14.2%**

This is close to the observed coincidence of 16.9%.

SUPPORTING REFERENCES

1. Weimann, L., K. A. Ganzinger, J. McColl, K. L. Irvine, S. J. Davis, N. J. Gay, C. E. Bryant, and D. Klenerman. 2013. A Quantitative Comparison of Single-Dye Tracking Analysis Tools Using Monte Carlo Simulations. PLoS One 8.
